# Identifying indicators of aesthetics in the Great Barrier Reef for the purposes of management

**DOI:** 10.1371/journal.pone.0210196

**Published:** 2019-02-20

**Authors:** Nadine Marshall, Paul Marshall, Matt Curnock, Petina Pert, Adam Smith, Bernard Visperas

**Affiliations:** 1 CSIRO Land and Water/Oceans and Atmosphere, Townsville, Queensland, Australia; 2 Ecologic, Townsville, Queensland, Australia; 3 Pollinate, Sydney, New South Wales, Australia; Swansea University, UNITED KINGDOM

## Abstract

The aesthetic appreciation of natural places is one of the most fundamental ways in which people relate to their environment. It provides wellbeing, an opportunity for recreation and reflection, a sense of place, and cultural enrichment. It also motivates people to take care of natural places and to conserve them for current and future appreciation. Aesthetically valuable places also support significant economic activity. However, there is little guidance available to assist environmental managers and policy-makers to consider and integrate aesthetic values into decision-making processes. In this study, we present an approach for developing robust and practical indicators of aesthetic value to enable environmental managers to consider, assess and report on aesthetic condition and trend. We demonstrate its utility using the case of the Great Barrier Reef, a region currently undergoing significant social, economic and environmental change and an area formally protected, in part, for its aesthetic values. A qualitative scoping study with 30 key informants identified over 180 potential qualities contributing to reef aesthetics. We tested five for their utility in capturing key aspects of the coral reef aesthetic: (i) coral cover, (ii) coral pattern, (iii) coral topography, (iv) fish abundance, and (v) visibility. We asked 1,417 online Australians to aesthetically rate 50 out of 181 underwater coral reef images that varied in relation to these five attributes. Coral topography, fish abundance, and visibility were significantly correlated with aesthetic ratings, whilst coral cover and coral pattern were not. We also tested for demographic patterns in aesthetic ratings. Our pilot study has demonstrated that readily measurable characteristics of coral reefs can provide useful indicators of aesthetic quality, opening up opportunities for coral reef managers and policymakers to assess and track changes in aesthetics in ways that are relevant to the public. There is considerable scope to further advance capacity for monitoring and managing aesthetic values of coral reefs through additional research that resolves nuances in the meanings associated with aesthetics in coral reef settings.

## Introduction

Iconic natural places, such as those protected as World Heritage Areas, are famous for their beauty. The aesthetic values of these landscapes are often the major draw for visitors, and in many instances are the key driver for their inscription as protected areas. One of the world’s most well-known natural areas—the Great Barrier Reef–for example, was inscribed as World Heritage in 1981 for its “superlative natural beauty above and below the water” (http://whc.unesco.org/en/list/154). The importance of protecting beauty in nature has also become acknowledged in the context of sustainable development: the aesthetic values of biodiversity are recognised by Member States at the Rio+20 Conference as a part of the “Future We Want” (paragraph 197 [1]).

The significance of the aesthetics of landscapes is not a recent or superficial phenomenon. According to the ‘Savanna hypothesis’, there is a genetic basis for human preference for “beautiful” landscapes, driven by our sense that aesthetics is linked to attributes that also support fundamental needs such as gain and refuge [2–4]. In a more contemporary setting, beautiful landscapes provide wellbeing, an opportunity for recreation, cultural enrichment, cognitive development, reflection, a sense of place, and contribute to identity [5–12]. Their important contribution to well-being is derived through restorative effects, greater agreeableness, stress reduction, empathy, pro-sociality, and perspective taking [13–18]. Significantly, there are also important negative implications: a decline in aesthetic quality of natural landscapes reduces human wellbeing [19, 20].

Aesthetical values also translate to economic value, especially where they underpin significant nature-based recreation and tourism [21–23]. As an example of one of the most iconic nature-based tourism destinations in the world, the Great Barrier Reef illustrates the economic importance of natural beauty. The area is estimated to have a total economic value of $56 billion and to make an annual contribution of $6.4 billion to the Australian economy, which includes $2.2 billion of direct tourism expenditure per year [24, 25]. As the fastest growing sector of the world’s largest industry, nature-based tourism clearly provides a strong economic justification for targeted management of the aesthetics of many–if not all—protected natural areas [26].

Incorporating the aesthetic value of natural places into the objectives and practice of environmental management can not only maximise their contribution to human society, but also strengthen support for their protection [27]. The inspirational value of beautiful places has been long used by conservation advocates and environmental managers to generate and increase public support for the declaration of new protected areas [27, 28]. Incorporating aesthetic qualities into the set of values that are the explicit focus of natural resource management can also increase public support for ongoing management of these places.

Despite their importance, however, aesthetic values are rarely included in long-term monitoring programs–and are often not even explicitly included in management targets and objectives—of protected areas [29]. This makes it difficult for environmental managers to report against key performance indicators that relate to social, cultural and economic benefits derived from protection, and also to demonstrate maintenance of key values for which an area was given protected (or World Heritage) status.

While there are likely to be a range of historical and legacy reasons for the low profile of non-ecological indicators in natural resource management [30], it is likely that a key issue limiting the extent of reporting on aesthetics is the lack of appropriate tools and methods for measuring aesthetic values of natural landscapes. As an artistic, philosophical, and even mathematical endeavour [31], there have been efforts to understand and measure aesthetic properties. In particular, there have been significant efforts in urban landscapes and landscape aesthetics [32, 33]. However, tools for the routine and practicable assessment of beauty in protected natural areas have not received the same level of development. In this study, we explore the feasibility of defining indicators of aesthetic values that can be routinely and meaningfully applied in support of management of one of the world’s most valued natural areas, the Great Barrier Reef.

### Case study context

The Great Barrier Reef (GBR) of Australia is one of the world’s largest coral reef systems. It houses globally-significant biodiversity, a rich diversity of underwater habitats, and a scenic appeal that is universally recognised [34]. These values make it an international tourism attraction: in 2013, the Great Barrier Reef received an estimated 53.3M days of use, with 98% of all days comprising tourism visitation [23]. Tourism operators and commercial fishers are particularly appreciative of the natural beauty of the region, with the tourism industry alone worth over $5.2 billion each year [23]. It also holds immense value for local communities, where ‘beautiful’ is one of the first words that come to local residents’ minds when thinking of the GBR [23]. Residents and tourists seek opportunities to appreciate the natural aesthetics of the GBR through experiences such as diving and snorkelling, visiting beaches, boating and scenic flights [22]. However, like numerous reef systems around the world, the GBR faces several natural and human driven pressures that threaten both ecosystem health and aesthetic values [29, 34]. Ecological and environmental conditions on the GBR have been regularly monitored for decades in one of the most comprehensive long-term monitoring programs in the world [35]. This has revealed substantial declines in GBR health over the last decade, mostly attributed to cyclones, coral bleaching, declines in water quality, and outbreaks of crown-of-thorns starfish [35].

### Identifying robust indicators of aesthetic values

Academics have been assessing the aesthetic value of landscapes since the 1970s [6, 36]. Most of this work originates within terrestrial landscapes, but as Tribot et al. (2016) suggest, we can probably extrapolate into underwater landscapes. The aesthetics of underwater ecosystems are perhaps the least understood of all landscapes [6, 37], although we note the work of several others in this space [38–40]. In particular, we note Tribot et al.’s (2016) work on Mediterranean reefs, which found that negative aesthetic judgments of these reefs correlated highly with microbial abundance, or the health status of the system. They interpreted that people responded to less healthy images of reefs with ‘disgust’ or places that might be potentially harmful. They argued, like others (such as those associated with the Savanna hypothesis), that aesthetic preferences are innate and directed by human survival, where humans seek beneficial habitats that are functional and support human survival and wellbeing. However, humans have shared only a short evolutionary part of their history within underwater landscapes. Given that films, photos, paintings and media have all helped to connect a wider audience with underwater places [41, 42], it may be that people are transposing aesthetic cues from terrestrial environments towards underwater environments [6]. In identifying potential indicators to use within the GBR region, we look towards knowledge developed in terrestrial environments.

Within terrestrial environments, researchers have found that several natural cues inspire higher aesthetic scores. Rare species, diversity, biodiversity and complexity within landscapes are important [6, 43–46]. People prefer naturalness in its most generic form across a range of landscapes, where naturalness reflects the degree of wilderness, pristineness or state of minimum human influence [47–50]. Aesthetic appreciation is also influenced by composition, balance and harmony [51]. Colour can both contribute and detract from landscape aesthetics. According to the ecological valence theory of human colour preference, humans prefer colours strongly associated with objects that they like (e.g., blue is associated with clear skies and clean water) and dislike colours strongly associated with objects they dislike (e.g., brown is associated with faeces and rotten food) [6, 55]. Generally speaking, blue waters are preferred to yellow waters but yellow waters may be acceptable if they are perceived as "natural". Generally, colour saturation and colour brightness are positively correlated with aesthetic score. [52, 53].

Typically, across a range of landscape types, people prefer ‘openness, light and a good view’. Openness within a scenic environment refers to the degree of visibility and spaces within the surroundings and provides a sense of accessibility and movement [41]. This may relate back to our survival and evolution where women prefer large areas of openness and men prefer landscapes with some trees [54]. Scenic environments predictably include large areas of water, open blue skies or mountainous landscapes. Green areas congested with manmade objects such as buildings and roads are normally associated with lower ‘scenic-ness’ ratings [56, 57]. Within the context of underwater environments, Tribot et al. (2016), looking at reefs of Mediterranean France, found that that the most important traits influencing aesthetic preferences were related to shape.

Aesthetic ratings have also been related to congruency and continuity, or people’s sense of place, where familiarity as well as novelty are joint predictors of aesthetic judgment [58, 59]. The sense, or familiarity, that people have with a place can contribute to individuals’ aesthetic judgment of that place, where familiar places and landscapes tend to be perceived as more beautiful. However, novelty can also be important when people visit other places. From a tourists’ perspective, never-before seen landscapes are rated as particularly beautiful [45].

Aesthetics can also be experiential. The sound or smell that is associated with an image can be important when responding to visual cues. For example, the aesthetic of water might be influenced by the sound of its travel or by the flow of movement [60]. Similarly, social studies with survey scales of ‘clean-dirty’ attest the perceived hygienic and aesthetic conditions at a destination while scales such as ‘well-maintained-run-down’ emphasise the importance of upkeep of physical attributes [44, 51]. The ‘lively-peaceful’ scale highlights how tourists form aesthetic judgments based on the pace of sounds heard at a destination while ‘nature-made-human-made’ and ‘loud-quiet’ scales inform the importance of auditory cues and their source and volume [45]. In sum, aesthetic values are likely to be represented by a large gamut of indicators and influenced by a multitude of factors, and very little information or assistance exists to support environmental managers to develop monitoring programs to assess aesthetic condition and trend.

Indicators of aesthetic preferences within landscapes and seascapes need to be relatively objective and able to be similarly assessed by people through time to be useful for monitoring. It is likely that GBR managers are concerned that this might not be possible or sufficiently robust to assess for trends. For example, while some may judge an image by the pleasantness to the eye, another may regard its aesthetic value according to the composition of the image or its colour or light, whilst another may be influenced by the subject and an associated childhood experience [8, 61]. Datta et al. (2006) suggest that aesthetic preferences can be further influenced by meanings and can change with experience and knowledge. However Dinsdale (2009) showed that people similarly judged the status of coral reefs regardless of their previous knowledge or exposure. Similar assessments have also been supported by others such as Haas et al. (2015) working on Mediterranean reefs [21]. They found that human perceptions of aesthetics were sufficiently congruent to be used as an inexpensive monitoring tool for a reef ecosystem [21]. Our aim was to provide an initial attempt at identifying reliable indicators of aesthetic value to coral reef managers (of the GBR in our case), within the context of understanding the breadth of potential for such indicators, to determine whether outstanding universal values, underpinning the World Heritage Area listing, are being sustained or are degrading.

## Methods

### Qualitative study; key informant study

Our first aim was to scope the potential indicators of aesthetic value within the context of the Great Barrier Reef (GBR). Scoping was important to identify and compare the many ways in which people appreciated the GBR. Using our own established and local networks, we identified people who were generally recognised for their long-standing relationship with the GBR. They were 50% female, with a mean age of 52.8 years (range 42 to 72 years), and the participants included well-known local reef photographers, senior reef managers, famous reef experts (in ecological and social sciences), and senior tourism industry representatives. They were invited to participate in the study by the primary author via email and written consent was provided by return email by those interested in participating in the study. Most interviews occurred as face-to-face interviews and generally lasted less than 30 minutes. Two interviews were conducted by telephone. All interviews were conducted by the primary author and not audio recorded. Extensive notes were taken, instead. Ethics approval was received from CSIRO Human Ethics Committee (023/17). Due to potentially identifying information, data is available upon request due to ethical restrictions imposed by the authors’ institutional ethics committee. Requests may be sent to the chair of the ethics committee, Dr. Cathy Pitkin, at csshrec@csiro.au.

The interview questions were open-ended and broad in nature so as to capture the most comprehensive range of responses that we could. The questions included:

- What would you say are the important aspects of the GBR to protect, particularly those that relate to the aesthetic qualities of the Reef?
- What should we be monitoring in order to know whether these aspects are being protected?

Key themes and attributes identified from the interviews were analysed in Word, and text was coded into thematic concepts that could potentially be used as indicators (for example, colour, movement, glistening). All initial coding was undertaken by the primary author. This process generated an initial list of 184 potential indicators. Related themes and attributes were then merged through iterative analysis, and in conjunction with the authorship team. For example, words such as shape, texture, composition were merged together into a theme called ‘composition’. This process continued until a final list of 12 indicators emerged that were faithful to the interview transcripts.

### Quantitative study

From the 12 indicators we selected five that reflected distinct attributes of coral reefs, were analogous to existing biophysical measures, could be easily recognised by reef managers, and could potentially be incorporated into existing monitoring programs. They were (i) coral cover, (ii) coral pattern, (iii) coral topography, (iv) fish abundance, and (v) visibility.

‘Coral cover’ represents the proportion of live coral that covers the substrate in a coral reef scene (e.g. ranging from 0% to >80%). ‘Coral pattern’ is likened to ‘coherence’ within the field of landscape aesthetics where a harmony arrangement within a landscape composition, such as unity in colour, shape, or texture, exists [11, 62]. ‘Coral topography’ refers to the three dimensional complexity and structures present in a scene (e.g. a scene may be a flat landscape with few features, or a complex of pillars, valleys, caves, etc.). ‘Fish abundance’ refers simply to the presence and abundance of fish in a scene, which may or may not include different species and sizes. ‘Visibility’ refers to the perceived clarity of the water, which influences the depth of perspective to the observer through the scene.

### Image selection

From a large pool of underwater photographs provided by co-authors and sourced from publicly available collections (e.g. https://gbrmpa.dams.me/), we selected 181 images representing typical, unglamourised, coral reef scenes. Selection criteria also included the perspective of a person in the water (snorkelling or scuba diving), looking forward across a substrate from a position between 5–10 metres above the substrate, in approximate depths of ten metres or less. This perspective was considered similar to the visual monitoring surveys conducted by the Australian Institute of Marine Sciences, the Great Barrier Reef Marine Park Authority, and other agencies. The photos can be accessed here: https://eatlas.org.au/nesp-twq-3/defining-assessing-monitoring-gbr-aesthetics-3-2-4

Members of the research team independently rated the 181 images against the five key attributes, assigning a rating of low, medium or high for each attribute, for each image. We note that this is a researchers’ point of view (across the biophysical and social sciences), and that non-researchers may have rated each attribute within each image differently. There was a high level of agreement between researchers, and in the small number of cases with differing scores, the image was reviewed and a final ranking agreed by consensus. The ranked images were mapped to a matrix to ensure each attribute and ranking were represented by a sufficient number of replicates to enable statistical analyses (see the full range of photos in S1 File). To address some smaller cell sizes in the matrix, some photos were duplicated, and a small number of images (n = 20) were edited using Adobe Photoshop^TM^ to add/remove, or enhance/de-emphasise certain attributes (e.g. fish and coral colonies were added/removed, contrast and sharpness were adjusted to give the effect of reduced water clarity).

#### Online survey

Photos were then delivered to an online service provider with the ability to reach a large number of random, representative Australians (www.pollinate.com.au). A survey was constructed to collect respondents’ basic demographic information, their self-rated level of interest in coral reefs, and their aesthetic ratings of a series of photos, using a scale of 1–10 (where 1 =“really unattractive”, and 10 =“really attractive”). Once an individual agreed to participate, by accepting to participate in the study, they were allocated a randomised selection of 50 photographs to rate from the pool of 181 photographs. Our pilot testing of the survey suggested that 50 photographs could be rated within a 10 minute period. The style of the survey was not dissimilar from some online games in which individuals are asked to rank their aesthetic preferences of fashion or interior design items (e.g. www.covetfashion.com, https://itunes.apple.com/us/app/design-home/id1010962391?mt=8).

A total of 1,417 individuals completed the online survey. Each photo was thus rated on the ten-point scale at least 380 times. Twenty-nine percent of the sample came from Queensland, and 71% were distributed across the rest of Australia. Some 62.3% of people came from Metropolitan Australia, whilst 37.7 came from rural/regional Australia. Some 51.4% were female. Participants reported a range of previous experience with the GBR and level of interest in coral reefs generally, where 7.2% had never visited (Table 1), and 7.9% did not find coral reefs particularly interesting (Table 2). Most participants (99.6%) were not part of a GBR based club or community groups (e.g. a fishing or diving club). Respondents’ mean age was 46.96 years (standard error = 0.47), ranged from 16 to 89. A copy of the dataset can be accessed by corresponding with the first author or Executive Manager, Social Responsibility and Ethics (csshrec@csiro.au).

**Table 1:** Level of experience with the Great Barrier Reef. Responses to the survey question, “Which of the following statements best applies to you?”.

		Frequency	Percent
	I have visited the Great Barrier Reef in the last 12 months	164	11.6
	I have visited the Great Barrier Reef–but it was more than 12 months ago	575	40.7
	I have never visited the Great Barrier Reef, but I would like to at some stage	572	40.5
	I have never visited the Great Barrier Reef, and don’t intend to	102	7.2
	Total	1413	100.0
Missing	System	4	
Total		1417	

**Table 2:** Responses to the survey question, ‘How would you best describe your interest in coral reefs?’.

		Frequency	Valid Percent
Valid	I generally find coral reefs not that interesting	111	7.9
	I generally find coral reefs interesting	767	54.3
	I generally find coral reefs really interesting	535	37.9
	Total	1413	100.0
Missing	System	4	
Total		1417	

Aesthetic ratings were analysed in SPSS, version 20, where we obtained the mean and standard error of the aesthetics rating for each photo. An analysis of covariance was conducted where (i) coral cover, (ii) coral pattern, (iii) coral topography, (iv) fish abundance, and (v) visibility, were tested in combination for their influence on the dependent variable, i.e. the mean aesthetic rating of each photo and each of the factors. The influence of gender, experience, age and self-reported interest in coral reefs were also assessed.

## Results

### Qualitative study; key informant study

**Fig 1:**
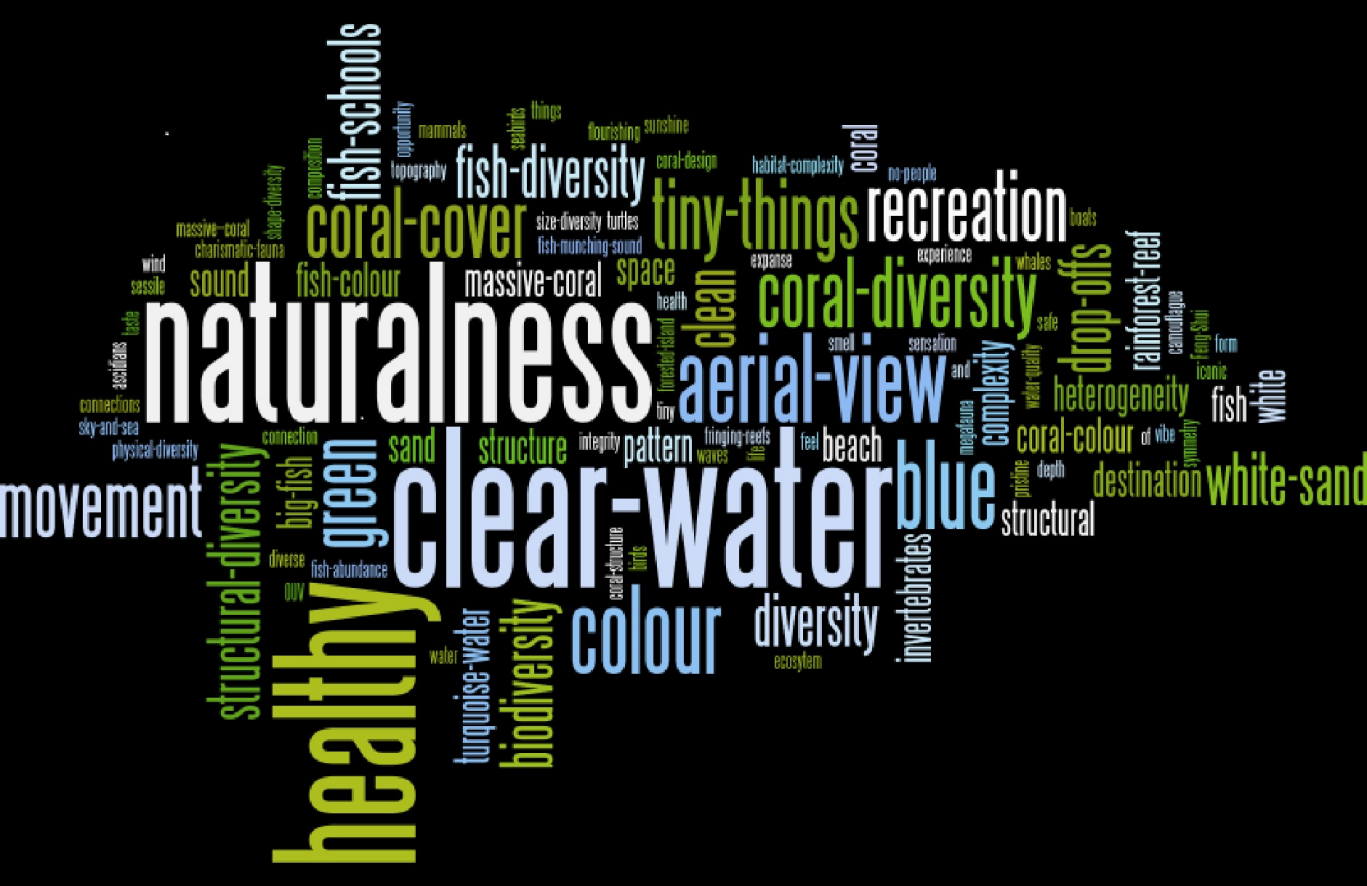
The full list of qualitative indicators, and the relative frequency with which they were mentioned by individuals. Larger words were mentioned relatively more often.

The interviews with 30 key informants identified over 180 potential elements or themes contributing to reef aesthetics, indicating the richness of meaning behind the concept of aesthetics, while also highlighting the challenge of distilling a complex characteristic into a set of robust and practicable indicators for monitoring purposes. The full list of qualitative indicators, and the relative frequency with which they were mentioned by individuals is represented in Fig 1 (the size of words reflects their relative frequency). Qualitative indicators were then coded by the author team into themes, as presented in the S2 File, where the verbatim list of indicators of aesthetic values, how they have been thematically coded, and categorised into the final list of 12 indicators, are presented. The final list of themes were: naturalness, composition, clear water, colour, charisma, coral, experience, tiny things, healthy, aerial, recreation, and fish. These are described in detail in Fig 1.

- **Naturalness**. Respondents spoke of the importance of naturalness in describing the aesthetic of the GBR, and the need for it to remain natural in order for aesthetics values to remain intact. Whilst some respondents did mention that the presence of boats, divers or snorkelers on the water could be aesthetically pleasing (see ‘recreation’ below), for the most part any human-made construct was generally perceived as an impact on aesthetic quality. Jetties, marinas, harbours were described as potentially aesthetically interesting infrastructure, where one respondent suggested that if such infrastructure had to go into the World Heritage Area, then every effort should go into ensuring it was as aesthetically pleasing as possible.
- **Composition**. Some participants talked about the aesthetic qualities of the GBR in terms of the structural landscape (where interesting topography was rated very highly), texture, pattern, diversity, habitat-complexity, structural heterogeneity, design, size and/or shape. Some participants used words such as Feng Shui, balance, and symmetry to convey the appreciation of the visual experience. Others described how they associated their visual experience with the experience of adventure, excitement, mystery or interest. For example, swim-throughs were mentioned twice as inviting images to explore.
- **Clear Water**. The clarity of the water was mentioned by nearly all respondents as contributing to their aesthetic appreciation of the GBR. For some, the clear water itself was beautiful, particularly with the sun shining through it, and particularly if there were small fish reflecting the sunshine just below the surface. Two respondents mentioned that they were aware that clear water did not always mean safe water where a cocktail of various toxins could exist within the water but not be visually detectable. Other respondents mentioned that muddy water was sometimes the more natural situation in areas such as Magnetic Island, and that knowing that helped them to appreciate water that was not necessarily clear.
- **Colour**. Colour was identified by many respondents as contributing to their appreciation of the aesthetic value of the Great Barrier Reef. Colour could refer to the colour of coral, the colour of coral bleaching, the colour of fish or the combination of colour within a single scene. Participants frequently described the blues, greens or turquoises of the water and/or water/sky and of the whiteness of the sand to describe important aspects of the GBR to protect. White sand visualised through clear blue water on a sunny day was mentioned several times.
- **Experience**. Some participants wanted to describe aspects of the GBR that they appreciated with words like movement (of fish or waves), connection with nature, destination, and referred to smell, sound, touch, taste, and/or opportunity for adventure and experience. These respondents regarded the aesthetics of the Great Barrier Reef in its entire sensory definition. For example, several participants described nooks and crannies, or swim-throughs or interesting topography that fell away to the unknown, as exciting or appealing and full of invitation to explore further. Aerial images of the Great Barrier Reef, with its channels and sea cays and islands, also made some participants want to experience the image.
- **Charismatic**. Whales, turtles, nesting seabirds, dolphins, big fish, clown fish, seahorses, nudibranchs, and several other charismatic animals were highlighted during the interviews as beautiful. Encounters with charismatic animals were described as special interactions that were very much about the personal response to being in nature and interacting closely with wildlife. These experiences were described as a critical part of their aesthetic appreciation of the GBR. One respondent remarked that the intense emotional environment created when interacting with Nature might be misinterpreted as beautiful, when it fact it might not be, but be a form of ‘love’ or ‘infatuation’.
- **Coral**. Coral was described by most people as the ultimate Great Barrier Reef aesthetic experience. Coral epitomised what was so special about the Reef and what was so beautiful. Coral was described in many different ways. Some respondents described it in terms of its composition (shape, topography, colour, texture, interest), whereas others described it in terms of abundance/cover and diversity of species and form. Two respondents made comment that coral reefs were like old growth forests, where massive Porites coral bommies represented the slow-growing ancient giants of the system, whilst the staghorns, whilst beautiful, represented the fast-growing weeds or undergrowth of the system, and that both forms together represented aesthetic values, and were together, related to coral reef health.
- **Fish**. Fish, like corals, were described either in terms of their physical presence (movement, colour, size, shape, reflection, sound, schooling), or in terms of their ecological value (fish abundance and fish diversity). Most respondents agreed that fish abundance and diversity in fish size were important contributions to their aesthetic experience below the water. One respondent described how she liked to imagine that there were large schools of various fish on the Reef.
- **Tiny things**. This aspect of aesthetic appreciation relates to below the water, and is closely related to the clusters, experience and charisma. Many respondents liked to describe how they enjoyed interacting with the Reef as part of their aesthetic enjoyment of the Reef. Inevitably, this involved aesthetically appreciating the small invertebrates existing amongst the coral such as the ascidians, nudibranchs, sessile organisms and worms. These tiny organisms were beautiful to look at in their own right, but were also beautiful in their contribution to the conglomerate of what a coral reef represented to them.
- **Healthy system**. Some respondents described what they thought was a beautiful experience both above and below the water in terms of words such as healthy, connectivity, whole-of-system, alive, vibrant. Respondents described how sometimes it wasn’t possible to tell whether a coral system or any other natural system was healthy or not, and that education played an important role in helping people understand what was healthy and what was not. One respondent suggested that it was possible to change one’s perspective on what was beautiful simply by informing them on whether it was healthy or not. Bleaching came up frequently as an example of a system that is unhealthy but beautiful. Another respondent wanted to point out that if the whole system was healthy (e.g. inshore, estuarine, coastal, mangroves etc.), then through the channels of connectivity, the best indicator would be a healthy coral reef (with coral, fish, invertebrates etc included). No-one described the role of people in the visual healthy image of a whole of Great Barrier Reef system.
- **Recreation**. Only a few respondents mentioned the role of people on the aesthetic of the Great Barrier Reef. This indicator cluster is closely related to the cluster of experience. These people described the image of boats on the water, particularly yachts, as relaxing, inspiring or beautiful. Importantly, they described only a few boats at a time, and sometimes they were described as nestled into bays. People enjoying themselves such as playing on the beach, and snorkelling or diving was seen as contributing to the aesthetic experience of these respondents.
- **Aerial view or spatial considerations**. Whilst most respondents were happy to discuss the aesthetic values of the Reef below or above the water, some respondents described the beauty of the reef from an aerial perspective. Several respondents described the appreciation of ‘space’. Vast, open areas that are so typical of the region were regarded by some as particularly stunning. This inevitably led to discussions about the various different scales that ought to be considered when one considers the beauty of the Reef. As such, this indicator cluster is closely related to tiny things and experience. The wide open space provided by beaches (Whitehaven and Port Douglas in particular) and the broad vista of the sea from the beach were particularly recognised as part of the aesthetic of the region.

### Quantitative study; Online survey results

**Fig 2:**
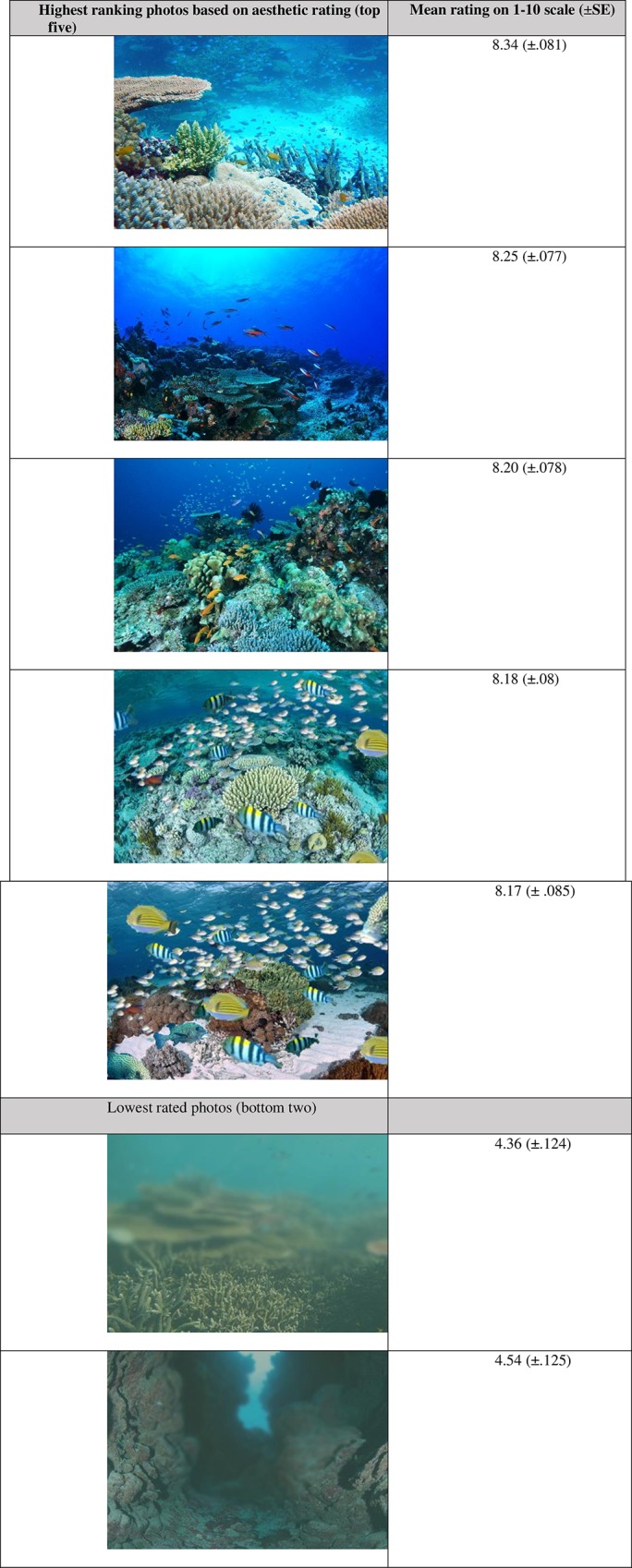
The highest and lowest rated photos by respondents of the online survey, including mean ratings and standard error scores. Note that the fourth and fifth favourite photo have been photo-shopped and are not ‘natural’.

The mean aesthetic rating for all 181 photos was 6.86, with means for individual images ranging between 4.35 and 8.34 (on a 1–10 scale) (see S1 File). The highest and lowest rated photographs are presented in Fig 2.

Results from the ANOVA suggested that coral topography, fish abundance and visibility were significantly correlated with survey participants’ overall ratings of the images’ aesthetic quality (Table 3). People who self-identified as having a high level of interest in coral reefs, and to some extent females, were also somewhat more likely to rate photographs higher than others (Table 4).

**Table 3:** Results of an Analysis of Variance (ANOVA) looking at the combined influence of factors on aesthetic ratings.

	Standard error of co- efficient	t	p
Coral cover	.079	.003	.997
Coral pattern	.076	-.668	.505
Coral topography	.070	2.089	.038*
Fish abundance	.057	7.362	.000*
Visibility	.119	5.401	.000*

Significance values less than 0.05 were regarded as significant and are identified by an asterisk (*). Interactions were not significant.

**Table 4:** Influence of demographic factors and a self-reporting ‘interest in coral reefs’ on aesthetic ratings of all 181 photos. Significant influences were regarded as those less than p = 0.05.

	F	p
Age	1.080	.307
Gender	3.983	.046
Visitation	1.657	.174
Interest in coral reefs	34.244	.000

## Discussion

Our pilot study has demonstrated that readily measurable characteristics of coral reefs can provide useful indicators of aesthetic quality, opening up opportunities for coral reef managers and policymakers to assess and track changes in aesthetics in ways that are relevant to the public. Results from this study suggest that there are numerous factors that could potentially be used to monitor aesthetic quality within the context of the Great Barrier Reef (GBR) Marine Park. However, not all factors are likely to be suitable indicators for monitoring, given their inherently subjective nature. ‘Reef structure’ and ‘reef health’, for instance, were mentioned by some interview respondents, and further work would be required to convert these into indicators that are similarly interpreted by all people. Our approach chose five indicators (attributes) that could potentially be used in objective terms for standardised monitoring and assessment of coral reef environments, and to quantitatively test them for their ability to predict a general observer’s assessment of coral reef aesthetics. We found that ‘coral cover’ and ‘coral pattern’, while important emergent attributes from our key informant interviews, were not reliably correlated with how people judged the aesthetic quality of coral reef images. It may be that elements contributing to ‘coral pattern’ are too numerous and/or subjectively appreciated, requiring additional interpretation. However, given that ‘coral cover’ is a clear, standardised and robust attribute, we were surprised that it did not correlate significantly with our respondents’ overall aesthetics ratings. We suggest that, in isolation, high levels of coral cover may be insufficient to appeal aesthetically to a general observer in the absence of other attributes (e.g. topographical structures, fish, and clear water). Coral cover (and pattern for that matter) may be appealing attributes in many situations, however other factors can combine to negate their overall attractive influence. Instances of reef flats with high coral cover that also coincided with low fish abundance and low visibility (or other cues) meant that people did not perceive them as particularly beautiful.

Coral topography, fish abundance and visibility were found to be statistically reliable predictors of aesthetic quality in this study. Coral topography, which provides habitat for creatures to hide in, might be appealing to people’s sense of adventure, exploration, and intrigue [27]. Such uncertainty and mystery could inspire a sense of curiosity in people’s minds, and elicit ‘potential experiences’, or ‘opportunities for exploration’ [9, 27, 63]. At the very least, topography contributes to more complex and interesting visual compositions in photographs.

The presence and abundance of fish were a highly significant influence on how people rated the underwater images. Fish abundance and fish diversity were both mentioned by most interviewees in the qualitative study. Fish may represent ecological completeness and wholesomeness within a seascape. One of our interviewees remarked “a coral reef without fish is like a playground without the laughter”. Fish also provide important, dynamic elements to a reef scene and photo composition. Fish add movement and colour, reflection and sound, and offer diversity of size and shape. Photos with high schooling behaviour (e.g. compare photo 173 with photo 174 and 175 in the S1 File) received very high aesthetic ratings, suggesting that it is possible that the arrangement and direction of schooling fish was particularly important in influencing aesthetic ratings. Two of our top five photos had been photo-shopped to over emphasise fish abundance, and given that such photo edits were obvious to us, this finding reinforcing the importance of fish as a key attribute of coral reef aesthetics.

Visibility, or water clarity, was also highly influential on ratings of aesthetic quality [61, 64–67]. Clear water has previously been found to increase visual satisfaction [64, 65, 68, 69]. People may have associated positive experiences with water condition, where experiences with clear water have been more positive than in less clear water (e.g. health issues, animal encounters). People may simply be responding to the blueness of the colour of the water, which evokes a sense of freshness and coolness, particularly on hot summer days [70].

Importantly, most demographic variables (including age, education, and previous visitation to the Great Barrier Reef) did not significantly influence aesthetic judgement. Apart from significantly higher overall ratings given by female respondents, which we are unable to explain, the only social factor that we found to be an important co-variable was the self-reported ‘interest in coral reefs’, where people who identified as more interested in coral reefs were more likely to rate a photograph highly. This can be interpreted where people are more likely to reinforce their own identity of being interested by finding the images of their interest particularly appealing.

## Conclusion

There is considerable scope to further advance the capacity for monitoring and managing aesthetic values of coral reefs, through research that resolves additional nuances in other attributes associated with aesthetics in coral reef settings. Many more indicators can and should be tested if aesthetic consideration, reporting and monitoring are to occur, not only within underwater seascapes, but within the variety of landscapes within which environmental managers work. This process of further testing is likely to be extensive, but with significant benefits for decision-making and management of coral reefs. Opportunities to perform mathematical image analysis may enable more reliable, reproducible and automatic measurements.

Within the Great Barrier Reef, we identify three steps that managers can take initially to manage for aesthetics within the region. Firstly, we suggest that aesthetic indicators can be trialled alongside established monitoring programs for the purposes of adaptive management. The indicators that we have tested are, to some extent, already being recorded in ecological monitoring programs, and they have the potential to provide additional insights into trend and condition of aesthetic value. However, it is unlikely that the three indicators that we have tested are alone sufficient for management agency reporting and assessment, and further work will be needed. We also suggest that by asking monitoring staff to include holistic ratings of the aesthetic quality of each site over time, it is likely that, whilst absolute measures will not be possible (given the low sample size of monitoring staff), aesthetic changes through time may be detectable, as other environmental values change. We have not considered issues of shifting baselines, nor have we tested for different cultural preferences of aesthetic quality (such as the extent to which indigenous residents have different aesthetic preferences in landscapes), but both are likely to be important, and will continue to be a source of concern in issues validity for those interested in implementing aesthetic monitoring. Understanding the influence of shifting baselines and diverse cultural preferences will impart greater confidence in monitoring processes. It is possible that as natural resources respond to the changing environment around them, the service of aesthetic quality is maintained (or the ecosystem service), whilst the indicators themselves (e.g. the fish and coral in the instance of the GBR) change.

Secondly, we see that environmental managers may be able to improve decision-making processes around developments in the Great Barrier Reef by including checklists around aesthetic impacts in the permitting process. Hass et al. (2015) developed an online tool called Sensiphi for converting the aesthetic appearance of an ecosystem in simple numbers that potentially provides a cost efficient monitoring tool (see http://www.sensiphi.com/). Their research showed that mathematical approaches to assessing compositional features of a coral reef digital image can correlate with human assessments of the aesthetics of the image. As computational learning algorithms improve (e.g. through AI), such tools are likely to produce results increasingly aligned with human assessments. However, thus far the relationship between coral reef health and the aesthetic appeal of such imagery has not been established empirically.

Thirdly, reporting on aesthetic values of the GBR to the UNESCO will become increasingly important if declines in the ecological state of GBR become more apparent. Managers of the GBR will be required to report on aesthetic changes as well as ecological and other changes, given the initial importance placed on aesthetic value. Cumulative impacts, for example, are inherently difficult to account for and manage, and it is difficult to allow some permittees permission to access the Marine Park whilst others do not. Aesthetic concerns are a potential way to consider both cumulative impacts as well as manage equity issues. Trade-offs between aesthetics and other objectives will need to be considered. It may be possible that the addition of aesthetics within a manager’s toolkit may contribute to delivering on multiple objectives. For example, economic demands on a natural resource could be refused to be permitted unless it could be shown that they do not trade-off on biodiversity, as well as aesthetic values. At this early stage, however, managers can refer to the three indicators used in this study to provide insights into the likely quality of aesthetic values, however, we suggest that further indicators are needed.

For many natural places, aesthetic values may be as important to protect as the natural values that normally dominate the formal objectives for environmental management [6, 10, 71]. Through our deep history and the modern experience, we now understand that beautiful natural places should be protected because they provide critical services to people [5]. In sum, there are many reasons to encourage environmental decision-makers to manage aesthetic values of protected areas. Aesthetically beautiful places are critically important for supporting local and international economies, human wellbeing, and stewardship. Such places provide the setting in which many people relate to nature, and for no other reason, the natural and aesthetic values of such places must be upheld. Environmental leaders require strong support from environmental and social scientists to enable them to assess and report on aesthetic condition and trend. Here, we provide three indicators that have the potential to be useful for this purpose.

## Supporting information

S1 File The 181 photos used in the quantitative study. (DOCX)

S2 File The verbatim list of indicators of aesthetic values, how they have been thematically coded, and categorised into the final list of 12 indicators. (DOCX)
