# Supplementary material for: Identifying indicators of aesthetics in the Great Barrier Reef for the purposes of management
Source: PLoS One. 2019 Feb 20;14(2):e0210196. doi: 10.1371/journal.pone.0210196 (PMC6382102; doi:10.1371/journal.pone.0210196)
Supplement: S1 File — (DOCX) [file pone.0210196.s001.docx]

# Supporting Information 1: The 181 photos used in the QUantitative study

**All 181 photos and their aesthetics, health and indicator ratings.** Aesthetics were rated on a scale of 1-10 (n~400 per photo from a pool of 1500 participants), (where 1=really, really ugly and 10= really, really beautiful). Health was rated by experts on a scale of 1-5 (where 1=very poor health and 5= excellent health). Indicators were rated by the research team on a scale of 1-3 (1=low, 2=medium, 3=high). Note that some photos are randomly duplicated, and duplicated in tandem. Note also that some photos have been photo-shopped to enhance, or detract, some features (one of the five indicators). Photos copyright Matt Curnock, Paul Marshall, GBRMPA.

| **#** | **Photograph** | **Aesthetic** | **Standard error** | **Health** | **Coral cover** | **Pattern** | **Topography** | **Fish abundance** | **Visibility** |
| --- | --- | --- | --- | --- | --- | --- | --- | --- | --- |
|  | 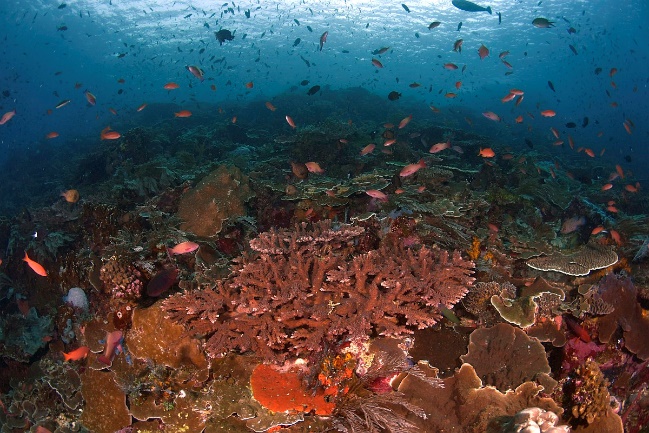 | 6.69 | .108 | 4.75 | 3.00 | 2.00 | 2.00 | 3.00 | 3.00 |
|  | 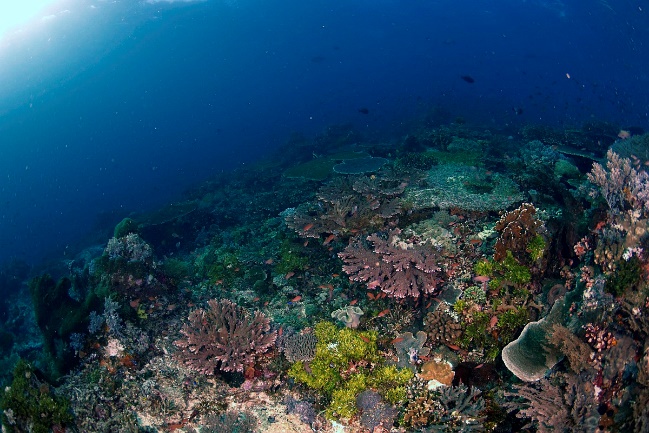 | 6.91 | .102 | 3.13 | 2.00 | 2.00 | 2.00 | 1.00 | 3.00 |
|  | 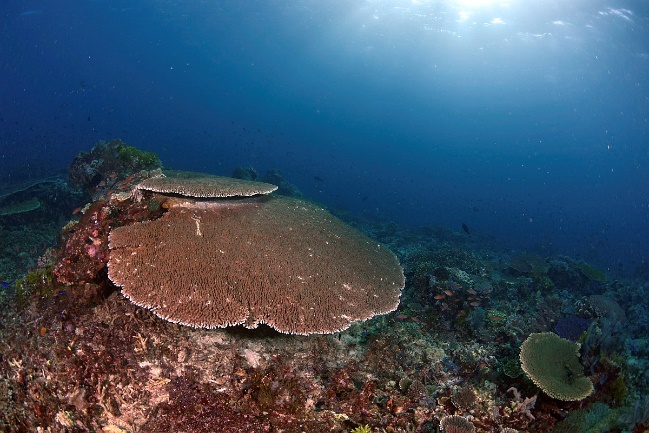 | 6.02 | .11 | 2.88 | 2.00 | 2.00 | 1.00 | 1.00 | 3.00 |
|  | 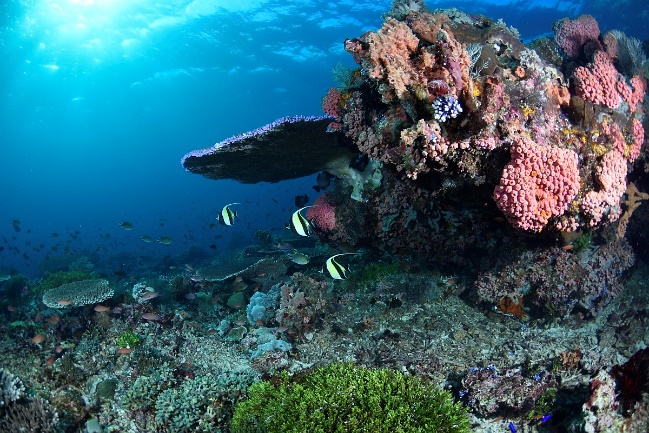 | 7.77 | .086 | 3.25 | 2.00 | 1.00 | 2.00 | 2.00 | 3.00 |
|  | 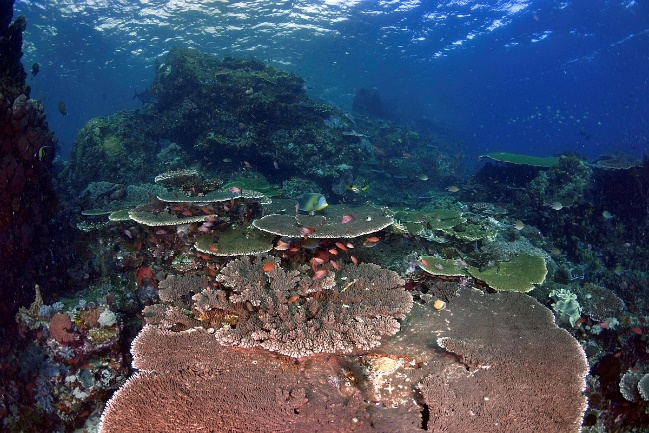 | 6.62 | .104 | 3.50 | 3.00 | 3.00 | 3.00 | 3.00 | 3.00 |
|  | 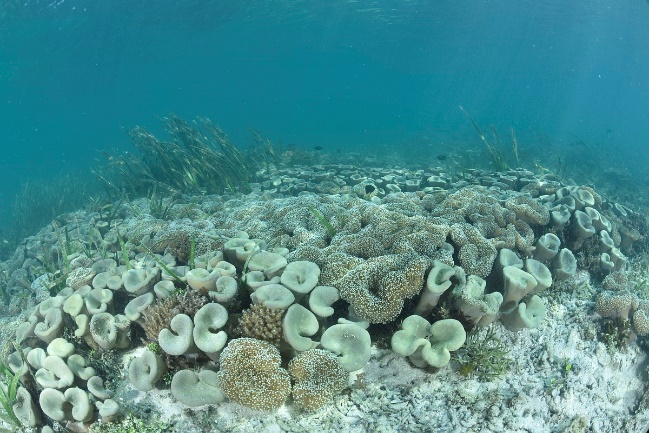 | 5.60 | .118 | 2.38 | 3.00 | 3.00 | 1.00 | 1.00 | 3.00 |
|  | 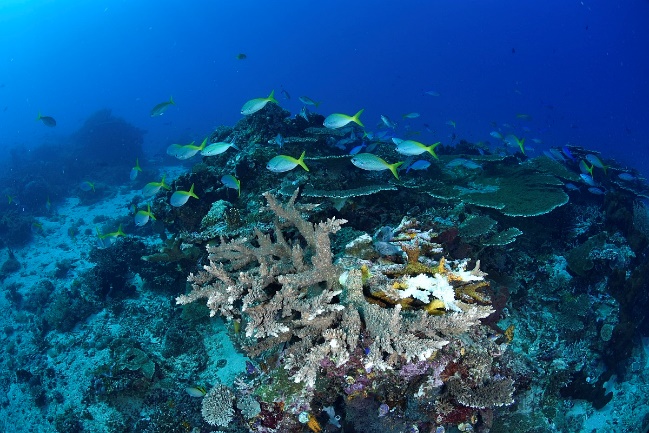 | 7.11 | .098 | 2.88 | 2.00 | 2.00 | 2.00 | 3.00 | 3.00 |
|  | 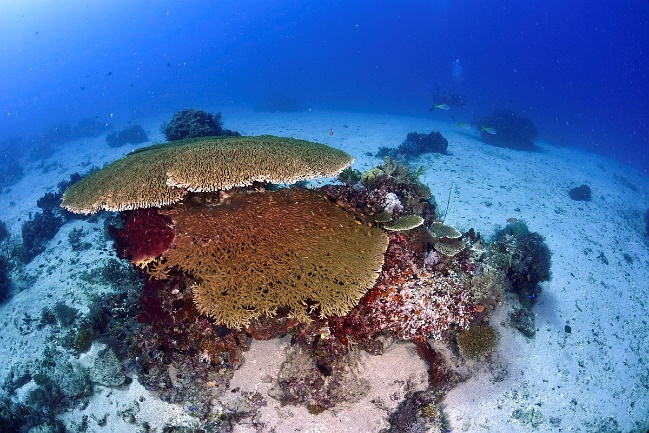 | 6.57 | .109 | 2.38 | 1.00 | 1.00 | 1.00 | 1.00 | 3.00 |
|  | 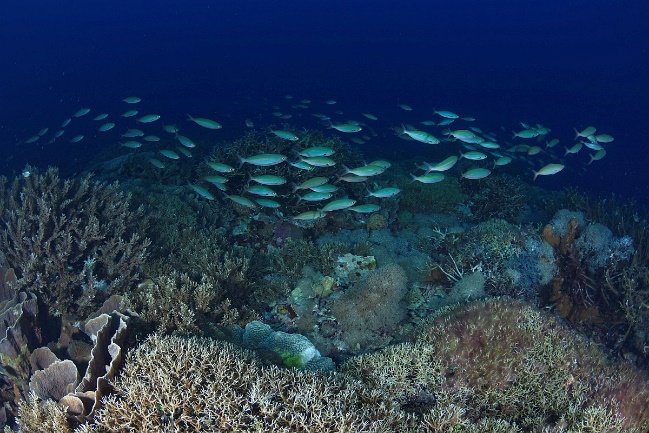 | 6.44 | .108 | 3.50 | 3.00 | 2.00 | 1.00 | 3.00 | 3.00 |
|  | 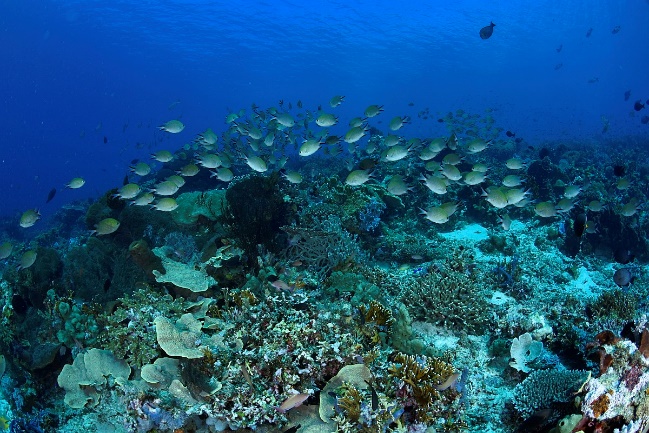 | 7.25 | .095 | 4.13 | 2.00 | 1.00 | 1.00 | 3.00 | 3.00 |
|  | 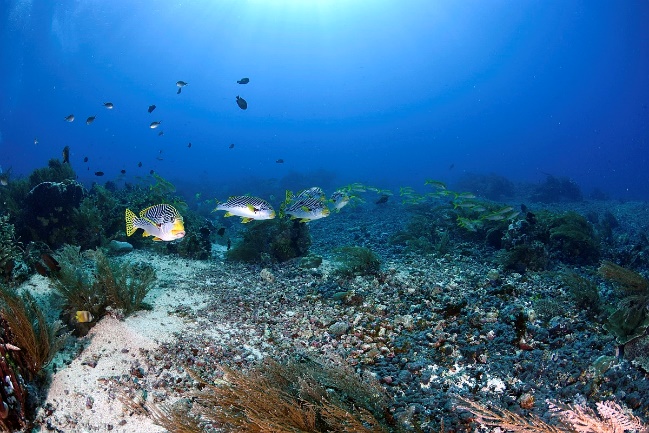 | 6.75 | .109 | 2.25 | 1.00 | 1.00 | 1.00 | 3.00 | 3.00 |
|  | 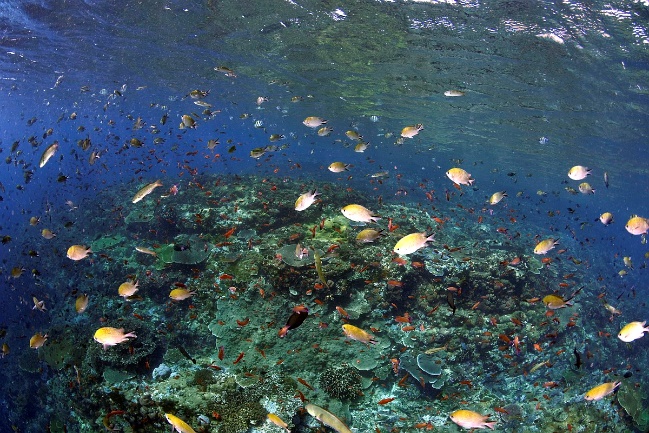 | 7.24 | .096 | 3.75 | 1.00 | 1.00 | 2.00 | 3.00 | 3.00 |
|  | 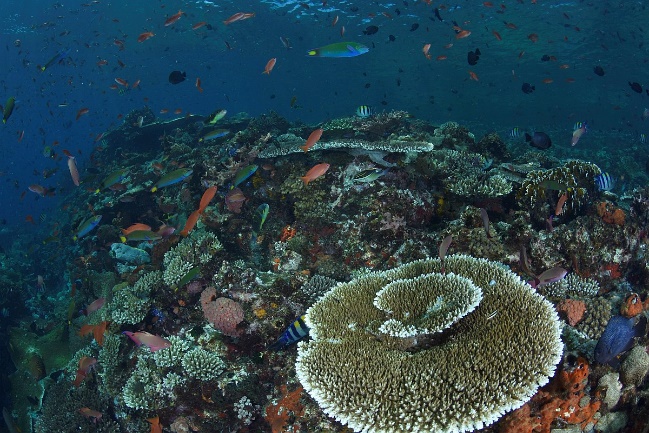 | 6.95 | .103 | 3.75 | 2.00 | 2.00 | 2.00 | 3.00 | 3.00 |
|  | 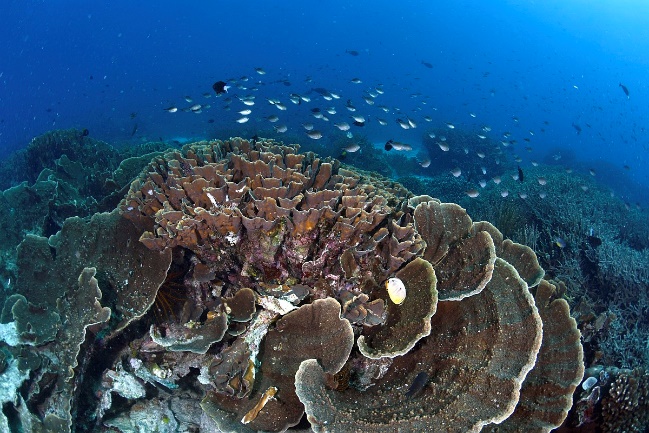 | 6.84 | .111 | 3.75 | 3.00 | 3.00 | 2.00 | 3.00 | 3.00 |
|  | 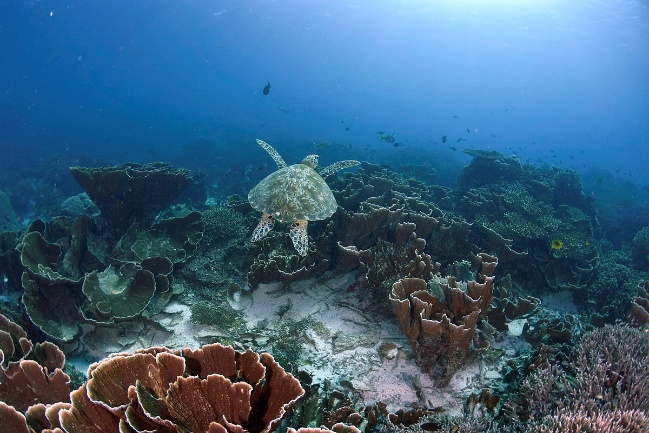 | 7.49 | .097 | 4.38 | 3.00 | 2.00 | 1.00 | 2.00 | 3.00 |
|  | 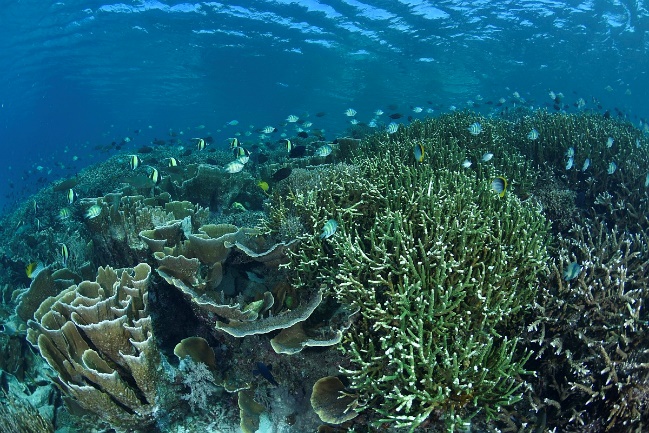 | 6.75 | .102 | 4.63 | 3.00 | 2.00 | 1.00 | 3.00 | 3.00 |
|  | 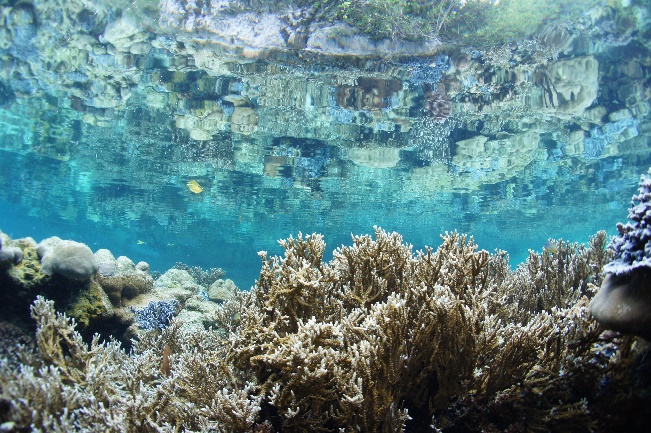 | 7.09 | .106 | 3.75 | 3.00 | 2.00 | 1.00 | 1.00 | 3.00 |
|  | 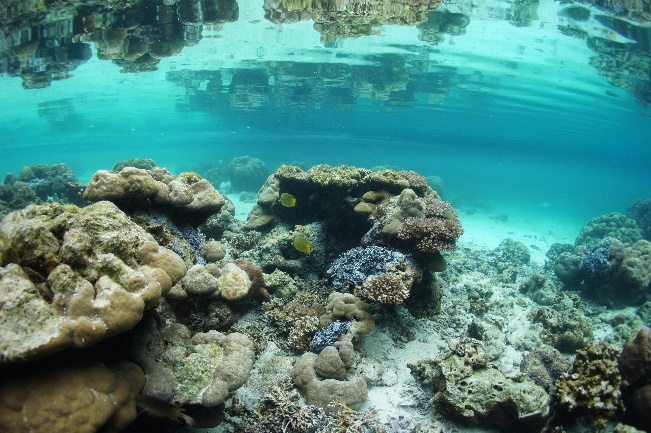 | 7.02 | .104 | 2.50 | 2.00 | 2.00 | 1.00 | 1.00 | 3.00 |
|  | 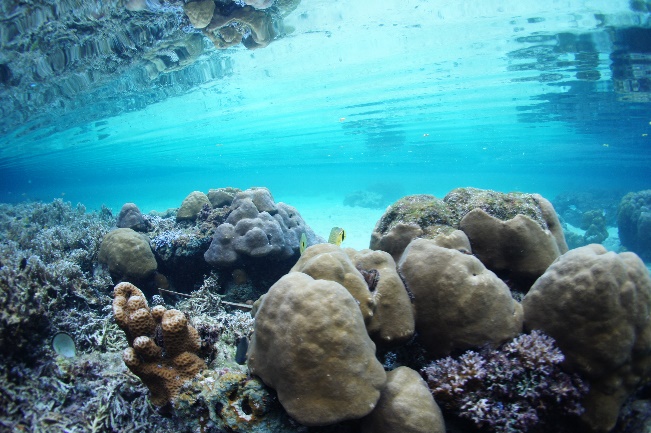 | 7.34 | .095 | 2.25 | 2.00 | 2.00 | 1.00 | 1.00 | 3.00 |
|  | 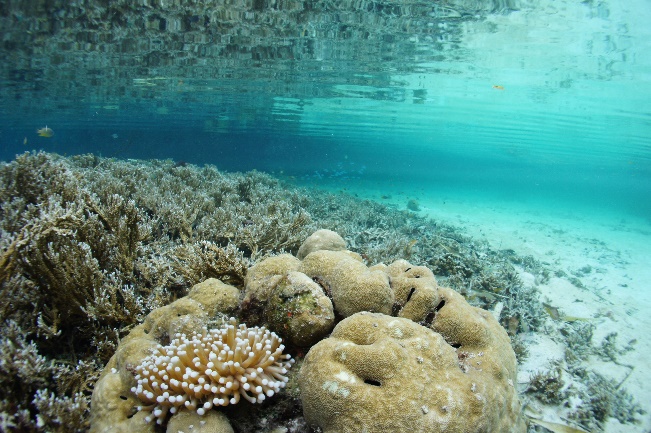 | 6.83 | .108 | 2.75 | 3.00 | 1.00 | 1.00 | 1.00 | 3.00 |
|  | 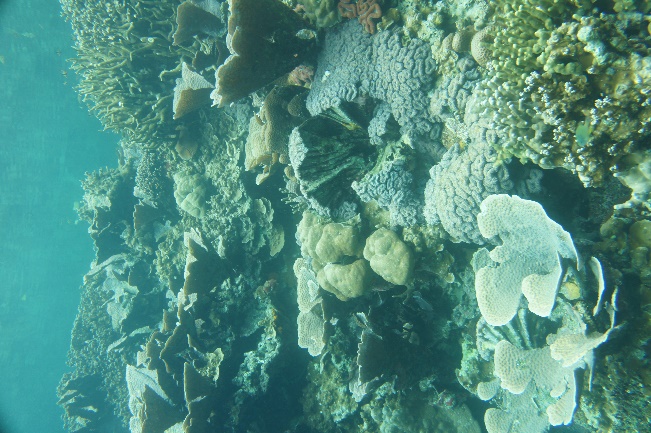 | 5.62 | .111 | 3.38 | 3.00 | 2.00 | 2.00 | 1.00 | 3.00 |
|  | 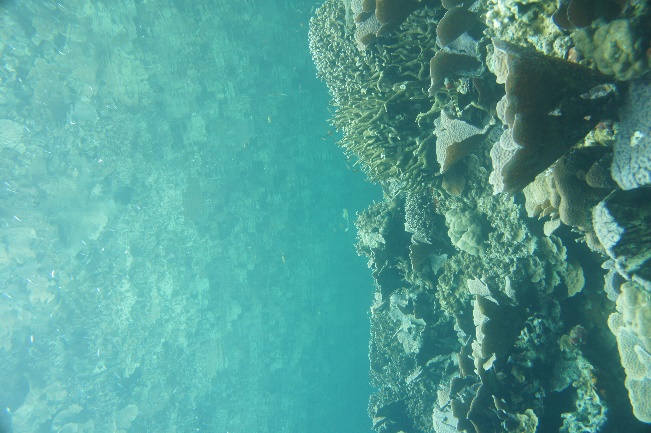 | 5.45 | .116 | 3.25 | 3.00 | 1.00 | 1.00 | 1.00 | 3.00 |
|  | 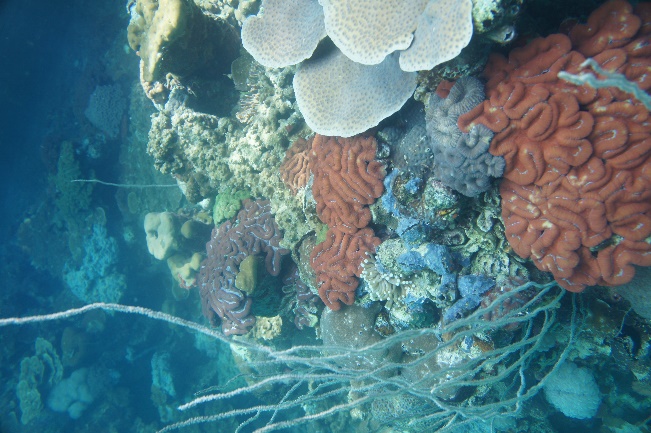 | 6.69 | .098 | 3.75 | 3.00 | 2.00 | 1.00 | 1.00 | 3.00 |
|  | 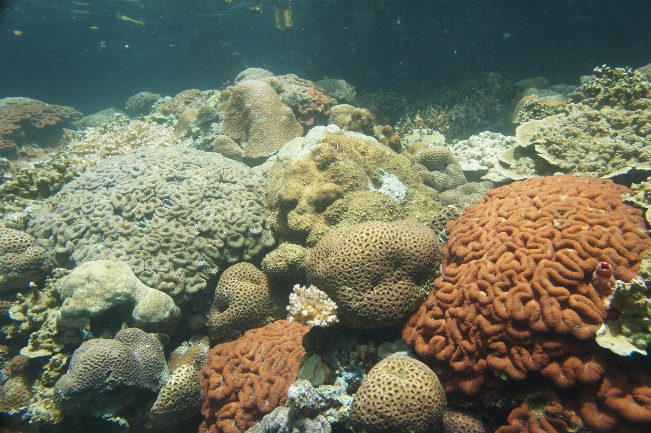 | 6.74 | .11 | 3.50 | 3.00 | 2.00 | 1.00 | 1.00 | 3.00 |
|  | 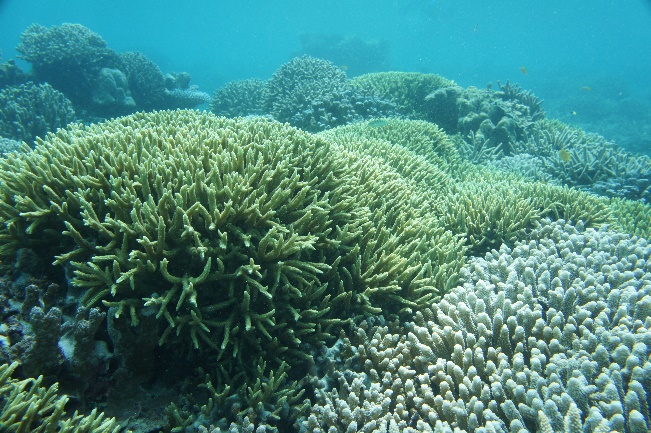 | 6.67 | .102 | 4.13 | 3.00 | 3.00 | 1.00 | 1.00 | 3.00 |
|  | 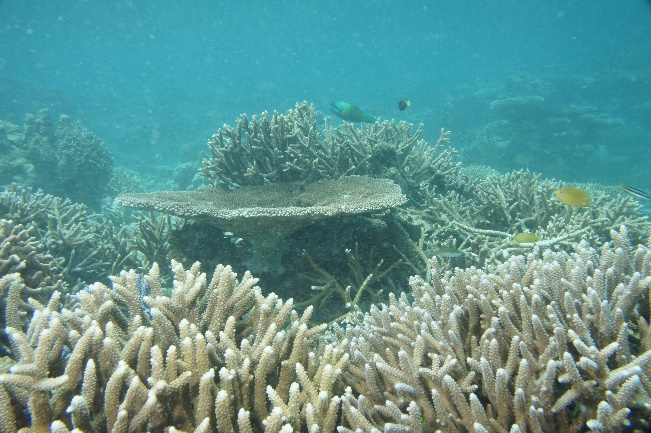 | 6.14 | .113 | 3.63 | 3.00 | 3.00 | 2.00 | 1.00 | 3.00 |
|  | 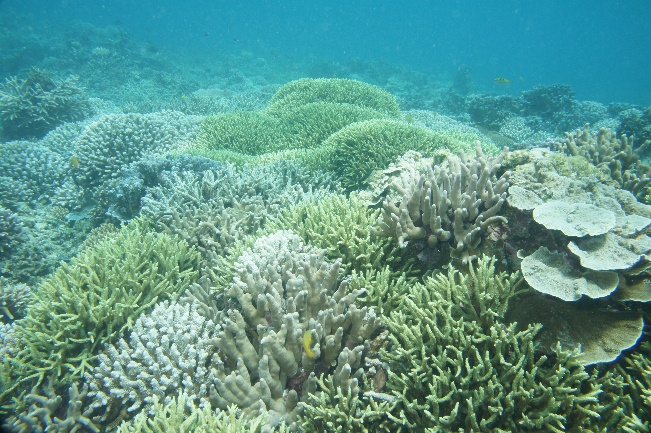 | 6.61 | .101 | 4.25 | 3.00 | 3.00 | 1.00 | 1.00 | 3.00 |
|  | 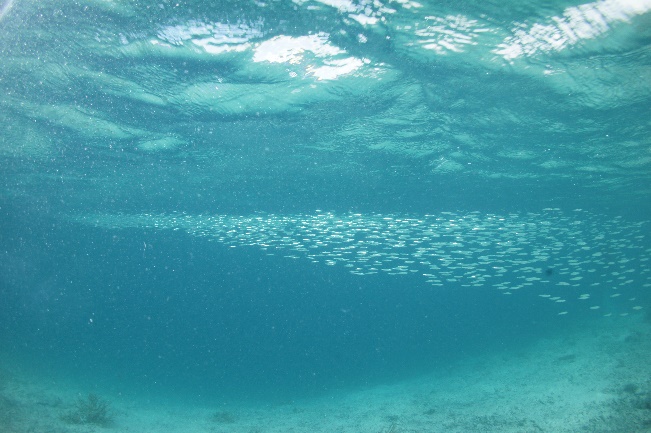 | 6.35 | .111 | 2.38 | 1.00 | 2.00 | 1.00 | 3.00 | 3.00 |
|  | 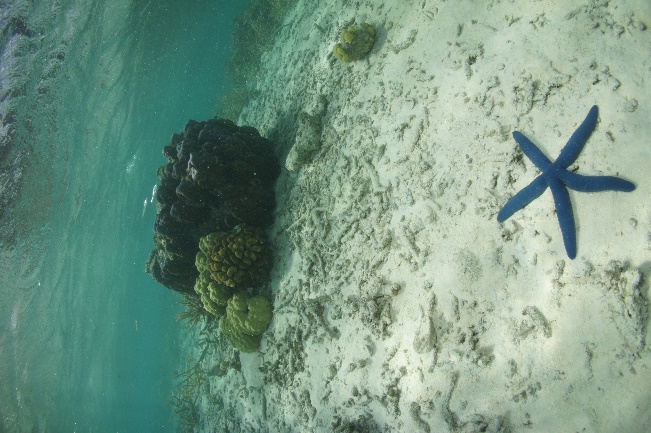 | 6.20 | .118 | 2.38 | 1.00 | 1.00 | 1.00 | 1.00 | 3.00 |
|  | 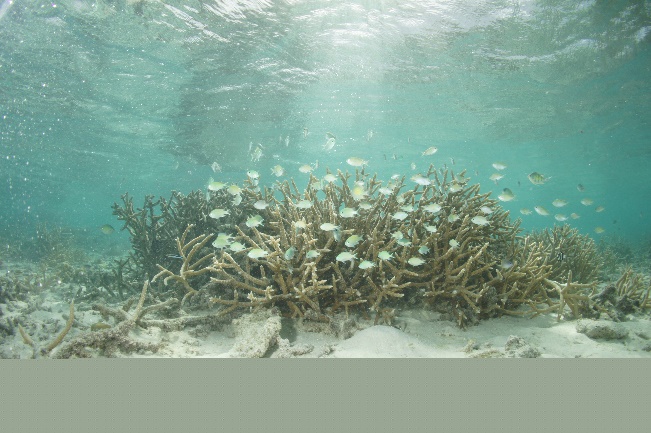 | 6.05 | .117 | 2.63 | 1.00 | 2.00 | 1.00 | 3.00 | 3.00 |
|  | 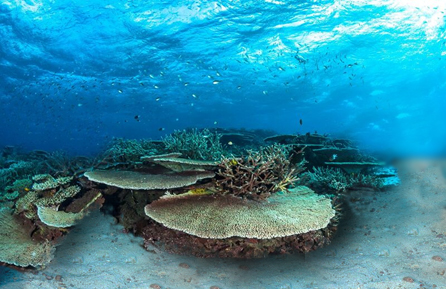 | 7.22 | .098 | 3.63 | 3.00 | 3.00 | 2.00 | 1.00 | 3.00 |
|  | 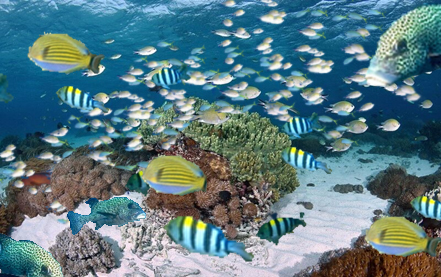 | 8.17 | .085 | 4.13 | 2.00 | 2.00 | 1.00 | 3.00 | 3.00 |
|  | 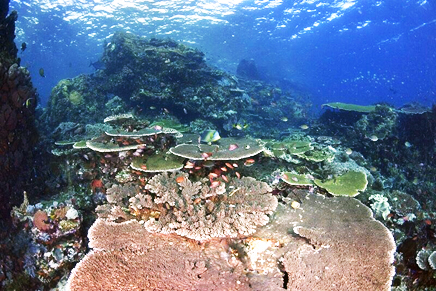 | 7.03 | .108 | 3.50 | 3.00 | 3.00 | 3.00 | 3.00 | 3.00 |
|  | 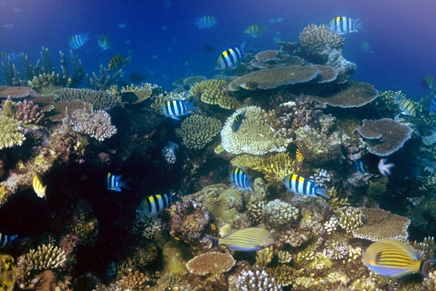 | 7.76 | .089 | 3.38 | 3.00 | 3.00 | 3.00 | 3.00 | 3.00 |
|  | 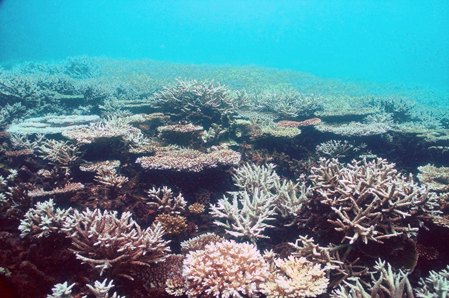 | 6.55 | .11 | 2.25 | 3.00 | 3.00 | 3.00 | 1.00 | 3.00 |
|  | 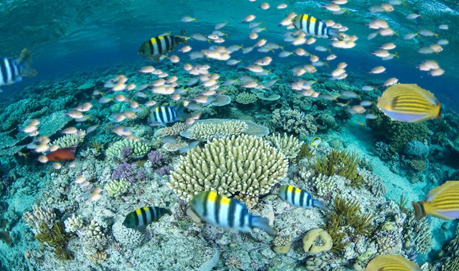 | 8.18 | .08 | 3.25 | 3.00 | 3.00 | 1.00 | 3.00 | 3.00 |
|  | 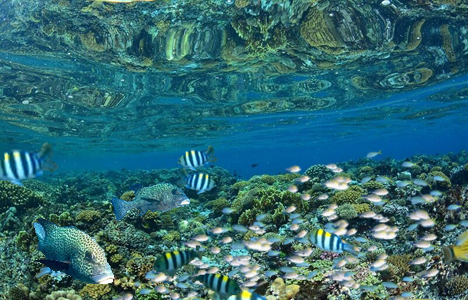 | 7.97 | .09 | 2.88 | 3.00 | 2.00 | 1.00 | 3.00 | 3.00 |
|  | 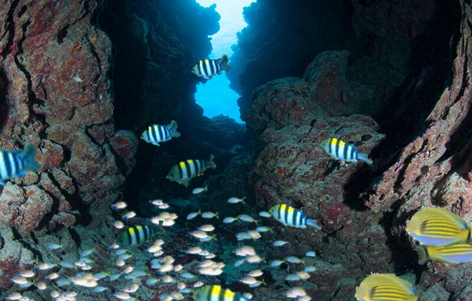 | 8.02 | .086 | 2.25 | 1.00 | 1.00 | 3.00 | 3.00 | 3.00 |
|  | 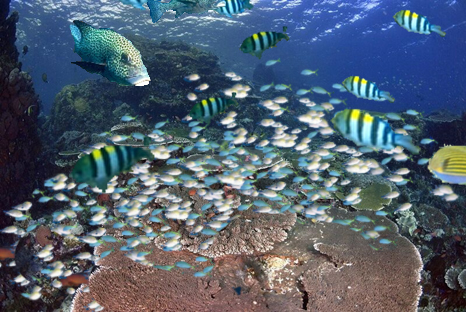 | 7.47 | .101 | 3.88 | 3.00 | 3.00 | 3.00 | 3.00 | 3.00 |
|  | 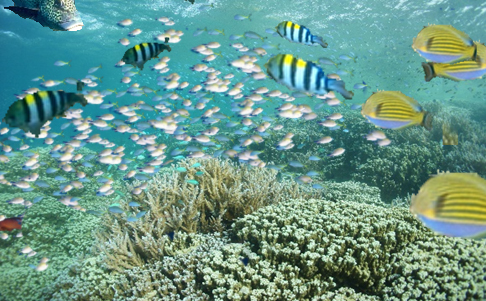 | 7.79 | .098 | 4.25 | 3.00 | 3.00 | 1.00 | 3.00 | 3.00 |
|  | 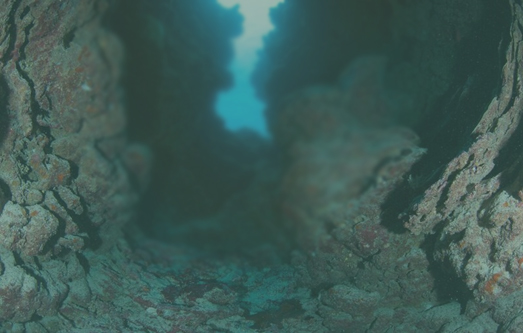 | 4.54 | .125 | 1.25 | 1.00 | 1.00 | 3.00 | 1.00 | 3.00 |
|  | 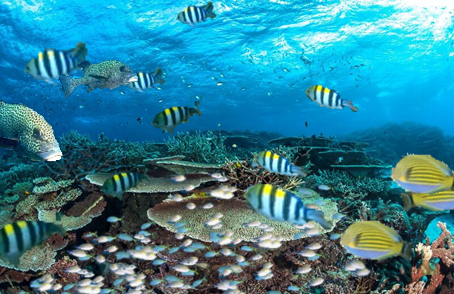 | 8.01 | .086 | 4.75 | 3.00 | 3.00 | 3.00 | 3.00 | 3.00 |
|  | 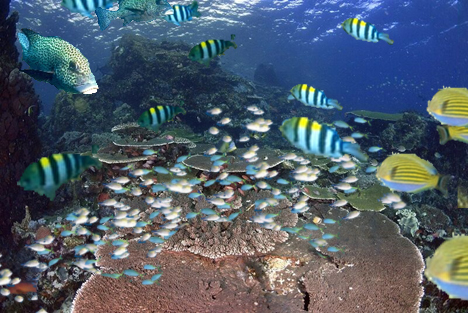 | 7.70 | .091 | 3.88 | 3.00 | 3.00 | 3.00 | 3.00 | 3.00 |
|  | 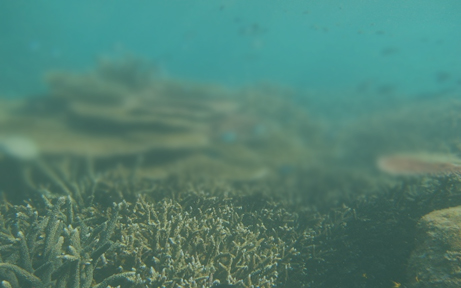 | 4.36 | .124 | 3.25 | 3.00 | 3.00 | 2.00 | 1.00 | 1.00 |
|  | 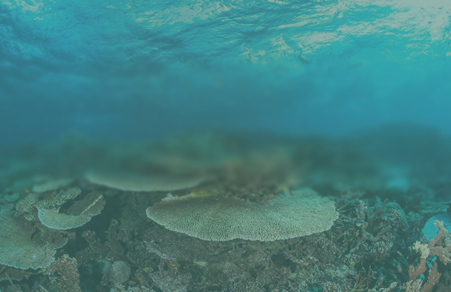 | 5.19 | .124 | 2.50 | 3.00 | 3.00 | 2.00 | 1.00 | 1.00 |
|  | 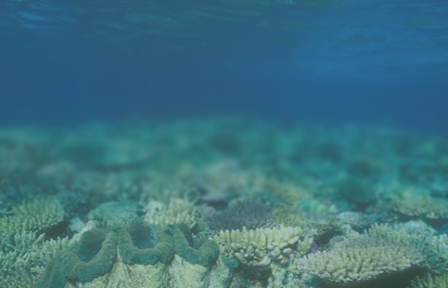 | 5.32 | .118 | 3.75 | 3.00 | 2.00 | 1.00 | 1.00 | 2.00 |
|  | 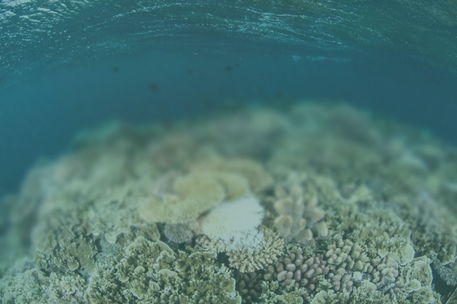 | 4.85 | .12 | 3.38 | 3.00 | 2.00 | 1.00 | 1.00 | 2.00 |
|  | 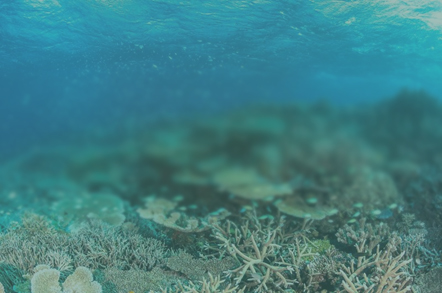 | 4.94 | .122 | 4.25 | 3.00 | 3.00 | 1.00 | 1.00 | 2.00 |
| 1. AS | 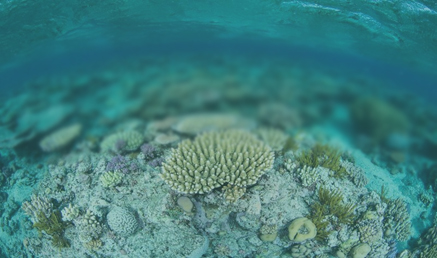 | 5.97 | .108 | 3.00 | 3.00 | 1.00 | 1.00 | 1.00 | 2.00 |
|  | 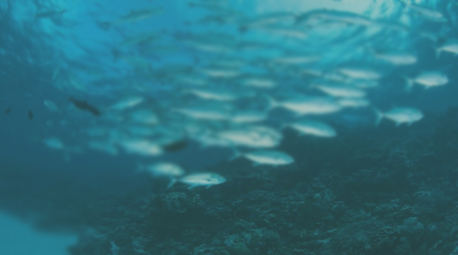 | 5.07 | .126 | 2.88 | 1.00 | 2.00 | 2.00 | 3.00 | 2.00 |
|  | 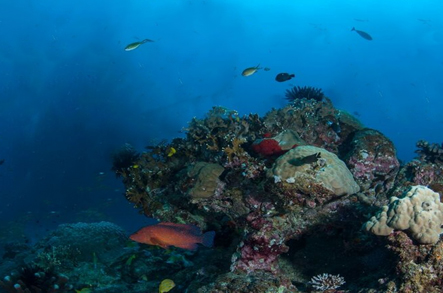 | 7.06 | .103 | 3.13 | 2.00 | 1.00 | 2.00 | 3.00 | 3.00 |
|  | 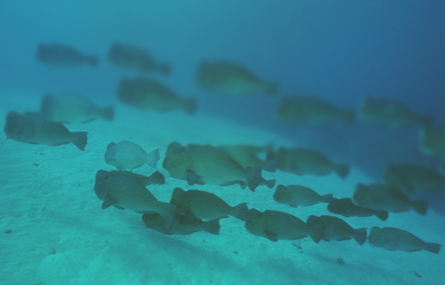 | 5.99 | .11 | 3.50 | 1.00 | 2.00 | 1.00 | 3.00 | 2.00 |
|  | 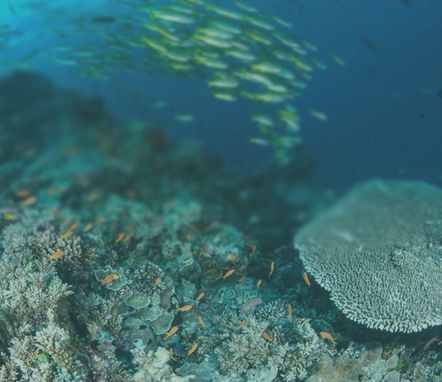 | 5.95 | .112 | 3.25 | 1.00 | 2.00 | 1.00 | 3.00 | 2.00 |
|  | 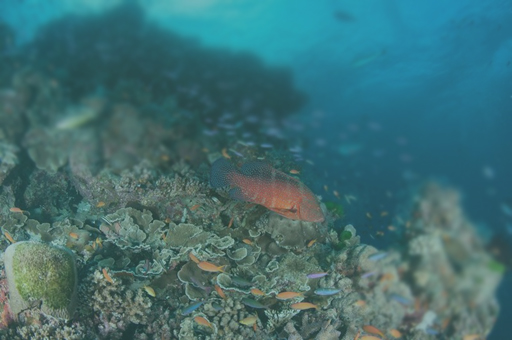 | 6.01 | .114 | 3.50 | 1.00 | 1.00 | 2.00 | 3.00 | 2.00 |
|  | 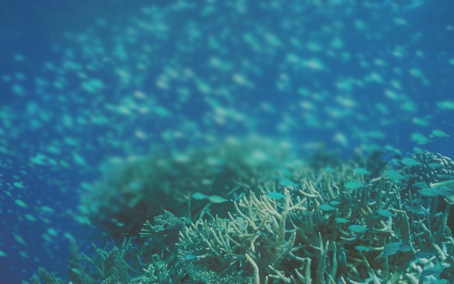 | 5.98 | .114 | 3.25 | 3.00 | 2.00 | 2.00 | 3.00 | 2.00 |
|  | 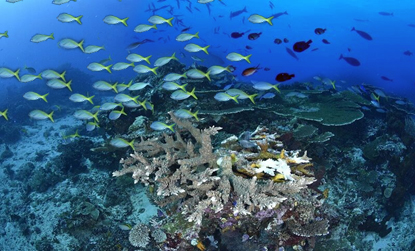 | 7.52 | .098 | 3.13 | 3.00 | 2.00 | 2.00 | 3.00 | 3.00 |
|  | 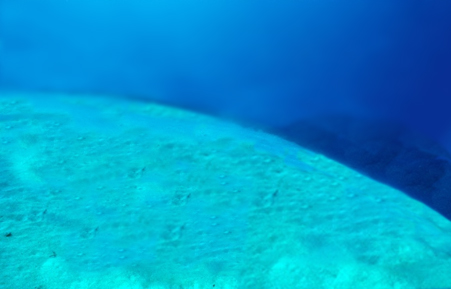 | 5.20 | .13 | 1.50 | 1.00 | 1.00 | 1.00 | 1.00 | 3.00 |
|  | 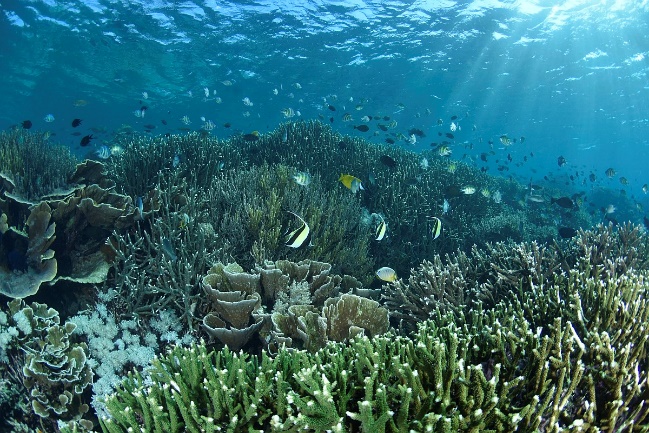  Please note: 58, 59, 60 are identical | 7.42 | .098 | 4.75 | 3.00 | 3.00 | 2.00 | 3.00 | 3.00 |
|  | 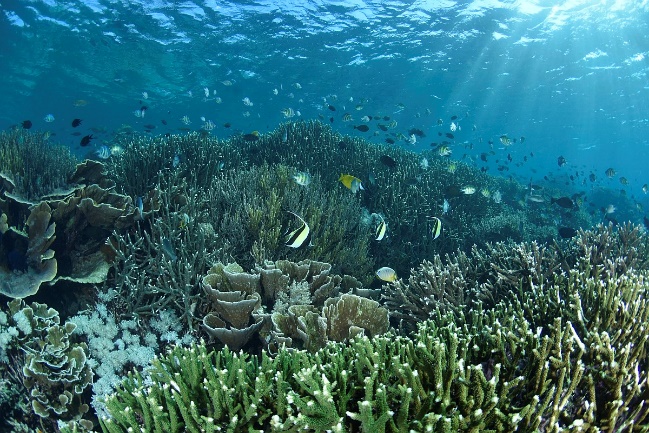 | 7.42 | .093 | 4.63 | 3.00 | 3.00 | 2.00 | 3.00 | 3.00 |
|  | 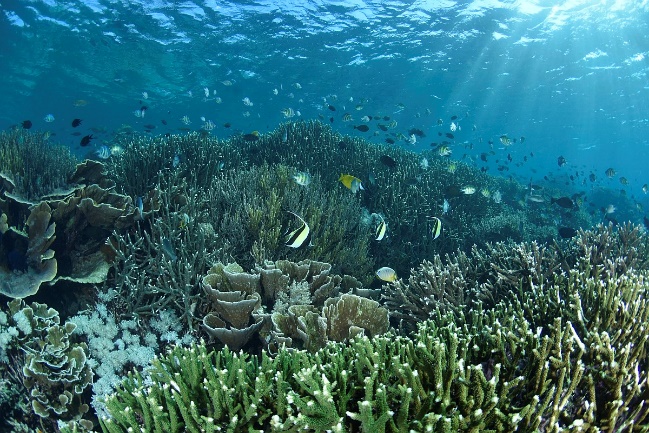 | 7.34 | .099 | 4.75 | 3.00 | 3.00 | 2.00 | 3.00 | 3.00 |
|  | 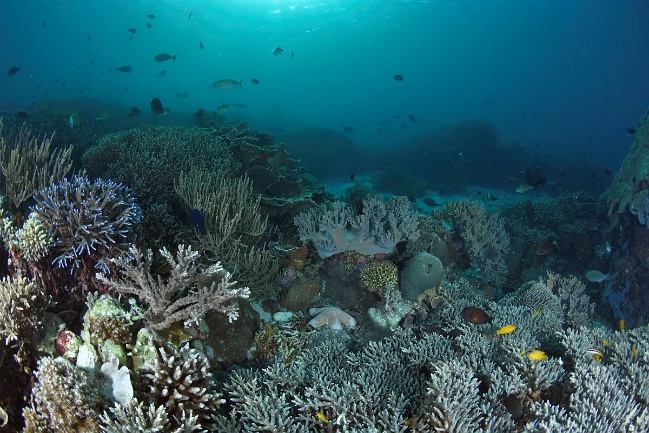 | 6.49 | .109 | 4.50 | 3.00 | 2.00 | 2.00 | 2.00 | 3.00 |
|  | 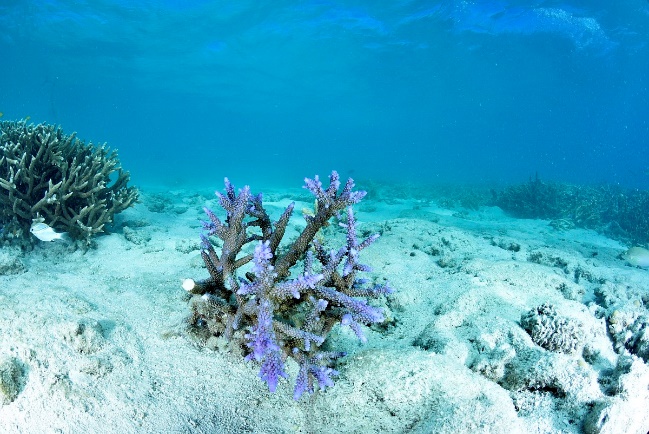 | 6.92 | .113 | 2.38 | 1.00 | 1.00 | 1.00 | 1.00 | 3.00 |
|  | 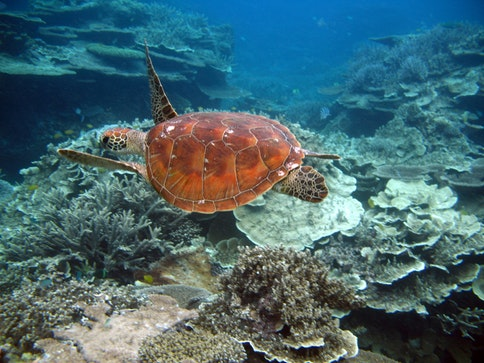 | 8.01 | .086 | 4.25 | 3.00 | 3.00 | 3.00 | 1.00 | 3.00 |
|  | 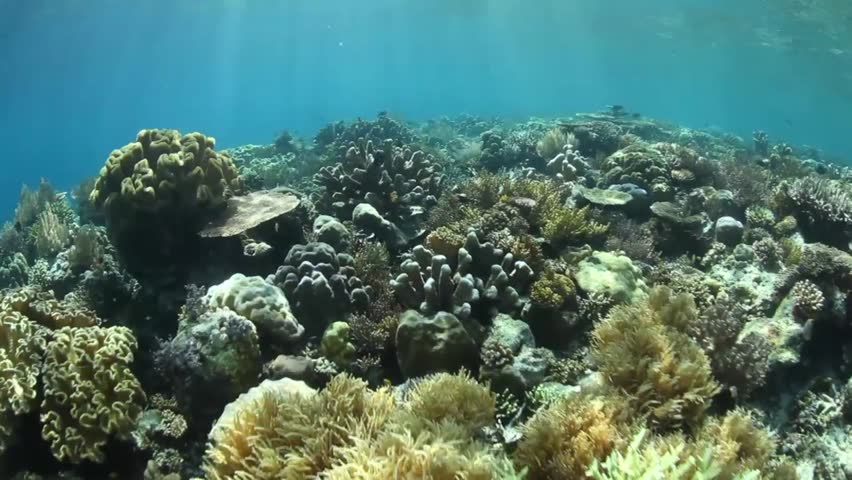 | 6.45 | .109 | 3.13 | 3.00 | 2.00 | 3.00 | 1.00 | 3.00 |
|  | 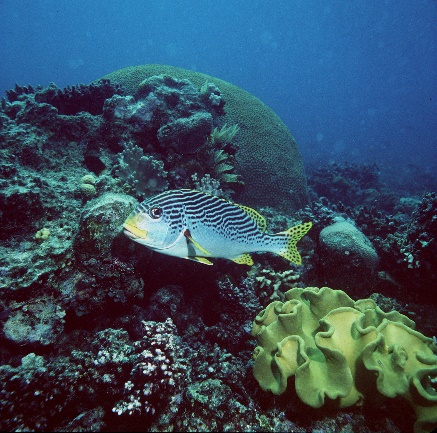 | 7.46 | .092 | 2.50 | 2.00 | 1.00 | 1.00 | 2.00 | 3.00 |
|  | 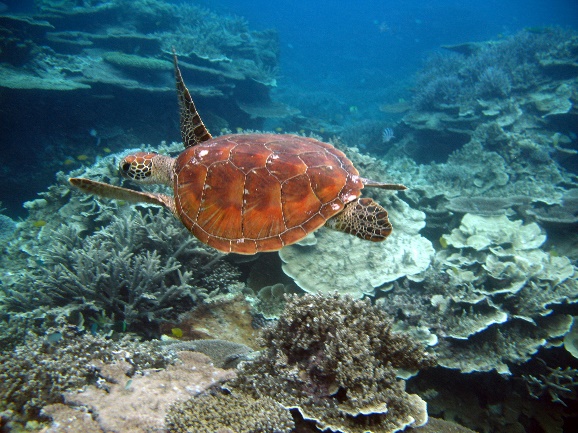 | 8.15 | .078 | 4.25 | 3.00 | 3.00 | 3.00 | 1.00 | 3.00 |
|  | 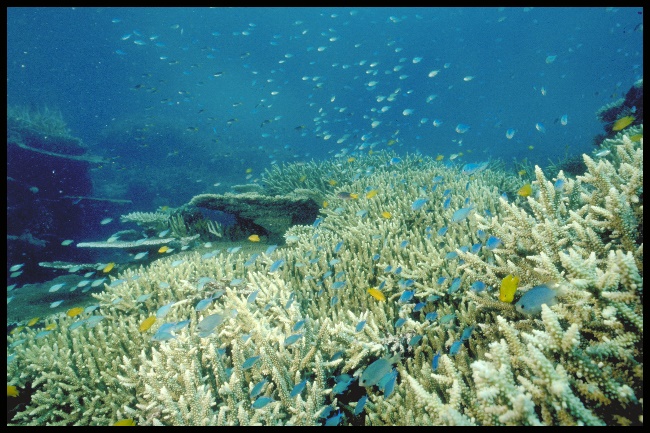 | 7.20 | .101 | 4.13 | 3.00 | 3.00 | 3.00 | 3.00 | 3.00 |
|  | 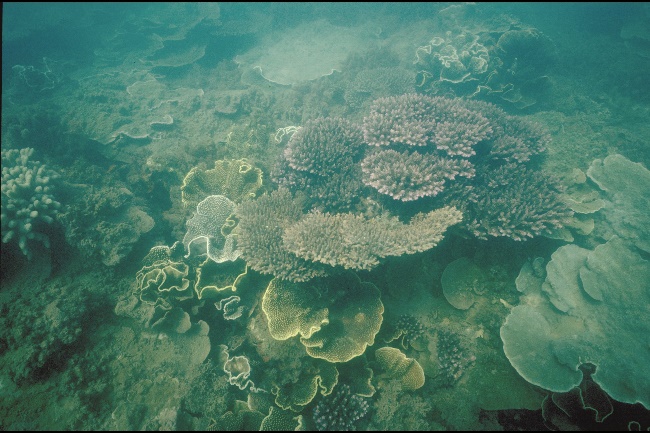 | 5.47 | .122 | 2.75 | 2.00 | 3.00 | 1.00 | 1.00 | 3.00 |
|  | 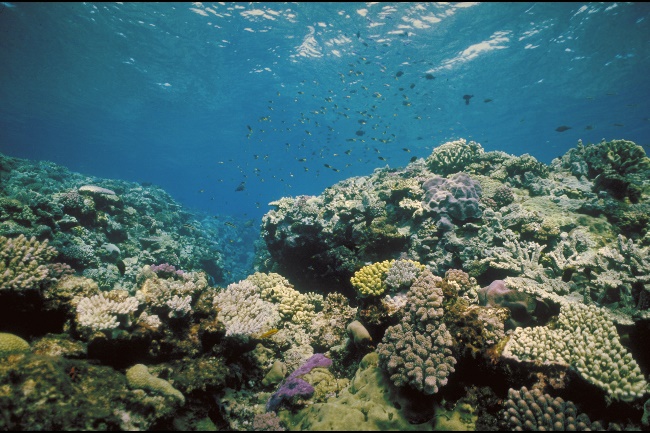 | 7.01 | .105 | 3.25 | 2.00 | 2.00 | 2.00 | 1.00 | 3.00 |
|  | 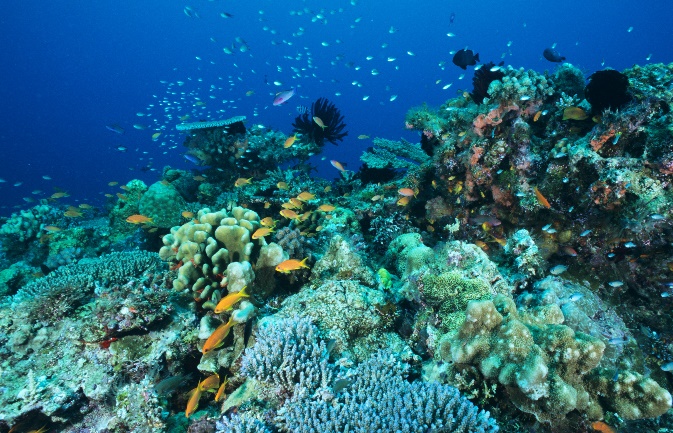 | 8.20 | .078 | 3.13 | 3.00 | 3.00 | 3.00 | 3.00 | 3.00 |
|  | 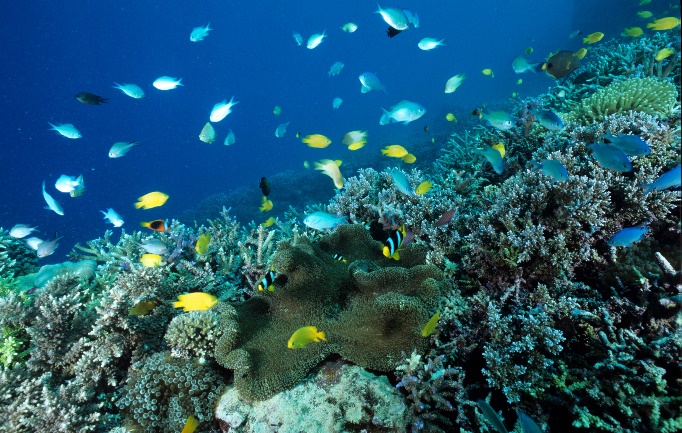 | 7.92 | .078 | 3.75 | 3.00 | 3.00 | 2.00 | 3.00 | 3.00 |
|  | 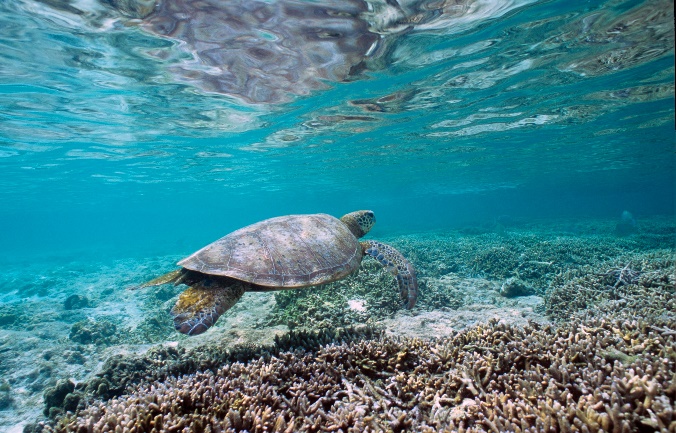 | 7.82 | .099 | 2.25 | 1.00 | 1.00 | 1.00 | 1.00 | 3.00 |
|  | 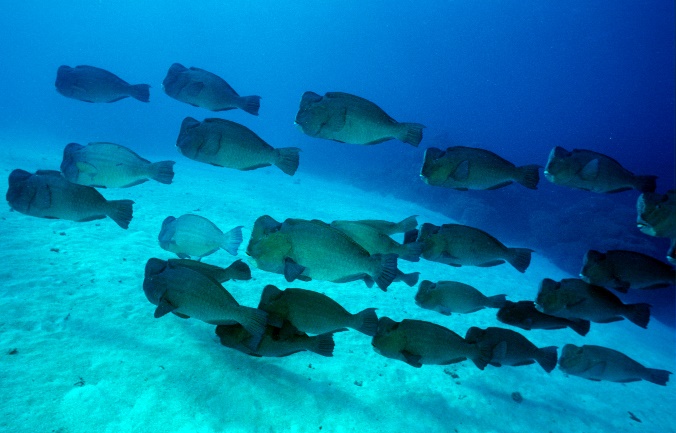 | 7.04 | .103 | 3.38 | 1.00 | 2.00 | 1.00 | 3.00 | 3.00 |
|  | 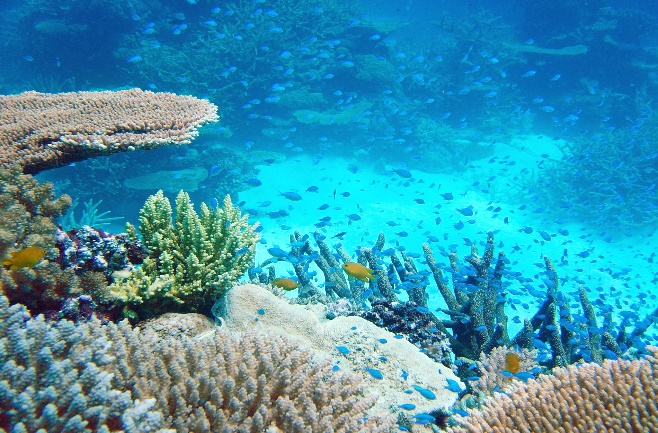 | 8.34 | .081 | 3.63 | 3.00 | 3.00 | 3.00 | 2.00 | 3.00 |
|  | 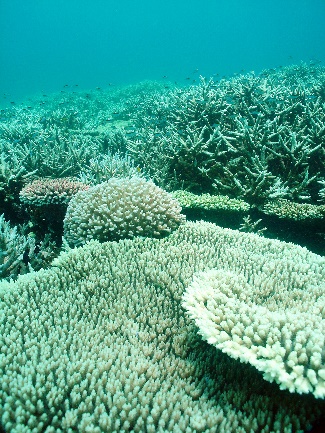 | 6.12 | .109 | 1.88 | 3.00 | 3.00 | 2.00 | 2.00 | 3.00 |
|  | 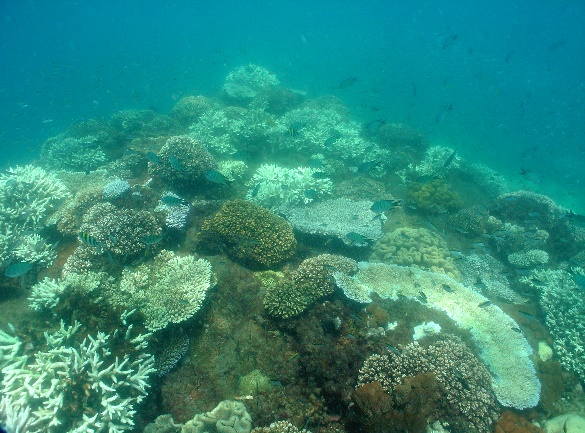 | 5.76 | .115 | 1.50 | 3.00 | 3.00 | 2.00 | 1.00 | 3.00 |
|  | 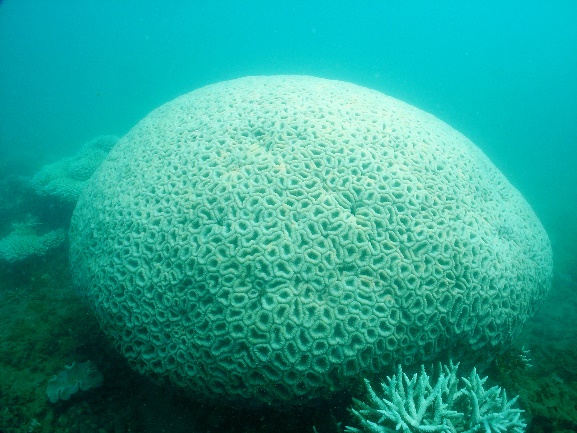 | 6.12 | .114 | 3.38 | 3.00 | 3.00 | 1.00 | 1.00 | 3.00 |
|  | 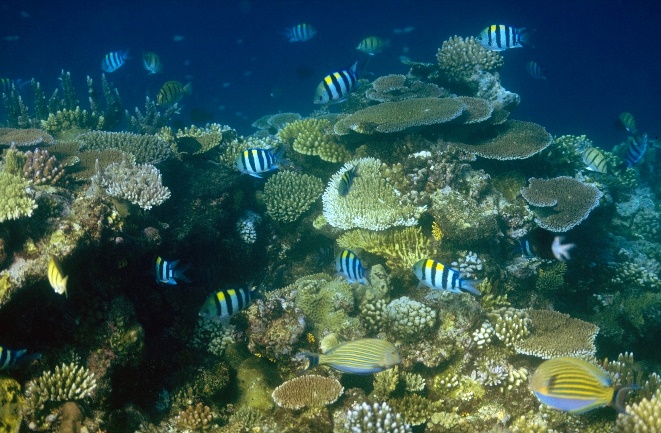 | 7.68 | .093 | 3.50 | 3.00 | 3.00 | 2.00 | 3.00 | 3.00 |
|  | 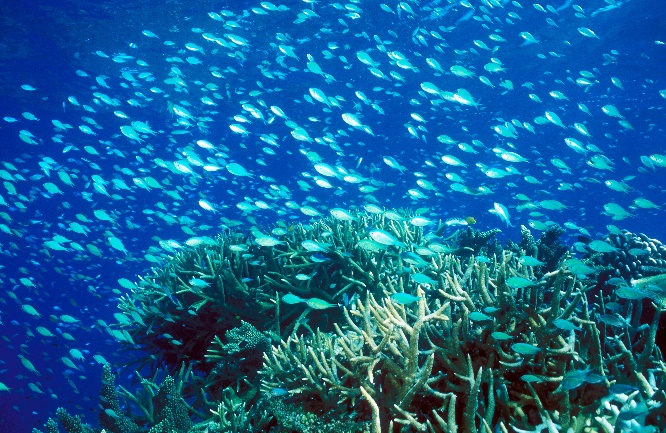 | 7.86 | .083 | 3.50 | 3.00 | 3.00 | 2.00 | 3.00 | 3.00 |
|  | 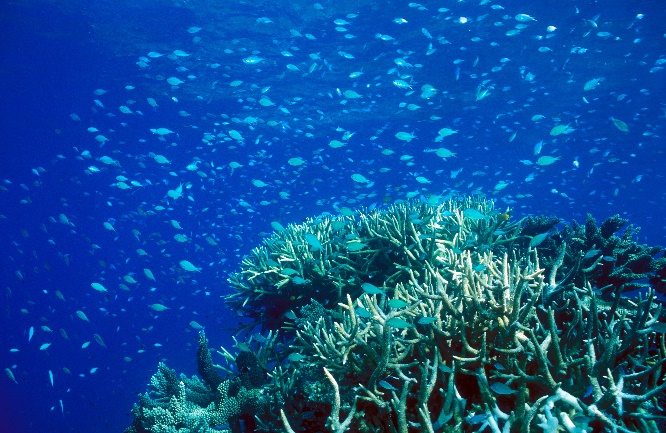 | 7.80 | .086 | 3.75 | 3.00 | 3.00 | 2.00 | 3.00 | 3.00 |
|  | 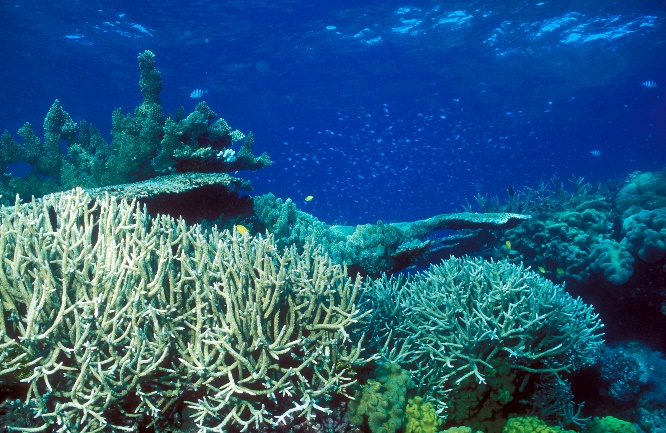 | 7.42 | .094 | 4.00 | 3.00 | 3.00 | 3.00 | 2.00 | 3.00 |
|  | 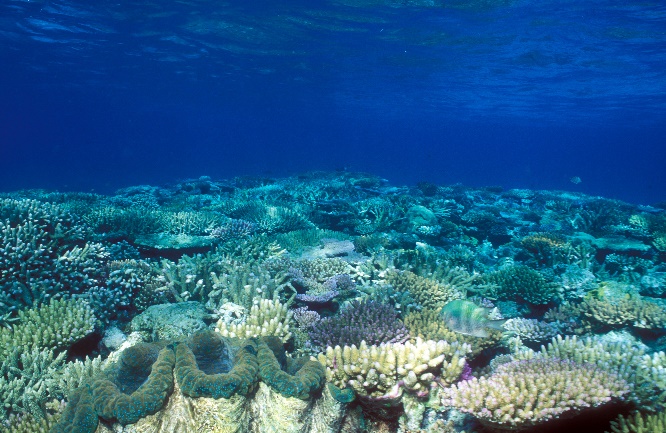 | 7.23 | .096 | 3.63 | 3.00 | 2.00 | 1.00 | 1.00 | 3.00 |
|  | 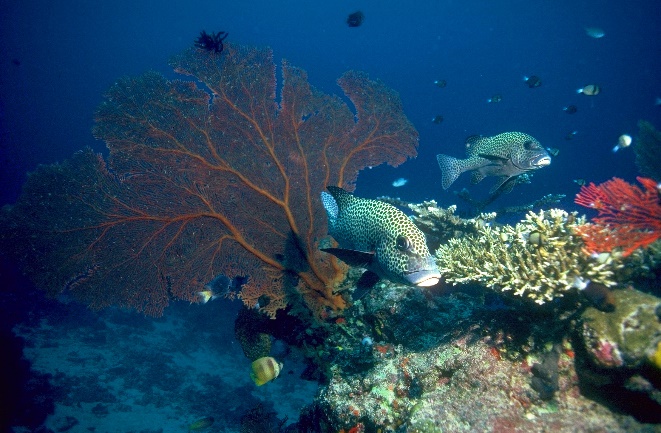 | 7.86 | .092 | 3.38 | 2.00 | 1.00 | 2.00 | 3.00 | 3.00 |
|  | 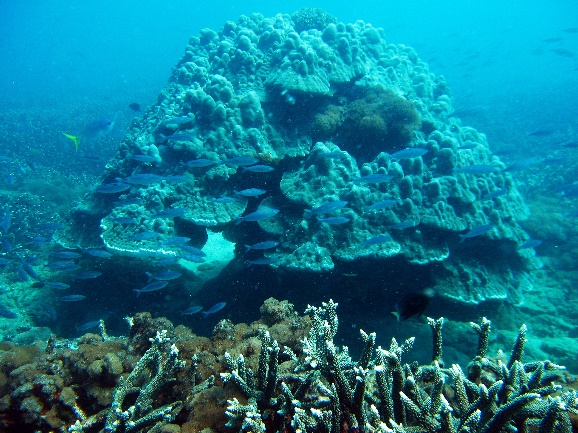 | 6.55 | .107 | 4.00 | 2.00 | 1.00 | 2.00 | 2.00 | 2.00 |
|  | 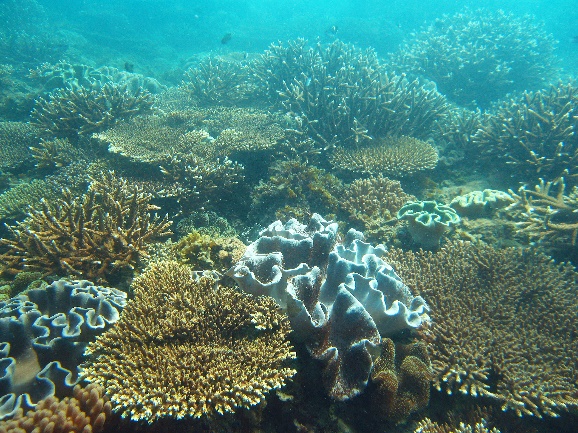 | 6.86 | .101 | 3.75 | 3.00 | 2.00 | 1.00 | 1.00 | 3.00 |
|  | 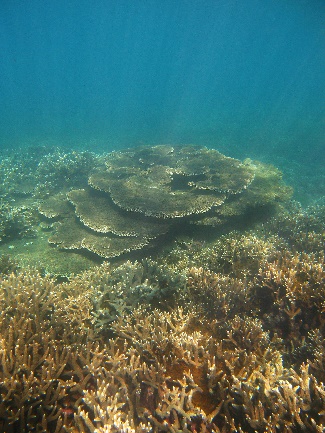 | 5.78 | .109 | 3.50 | 3.00 | 3.00 | 2.00 | 1.00 | 3.00 |
|  | 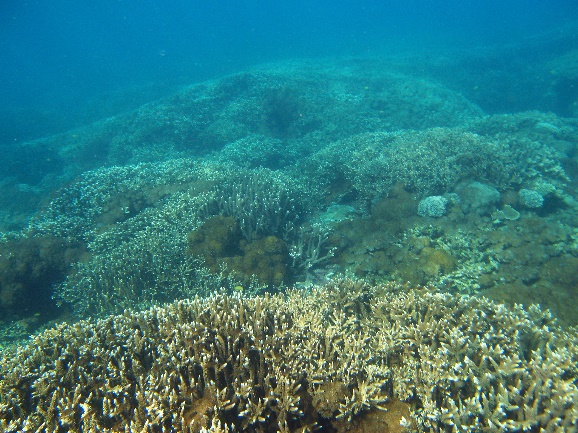 | 5.91 | .113 | 3.88 | 3.00 | 3.00 | 1.00 | 1.00 | 3.00 |
|  | 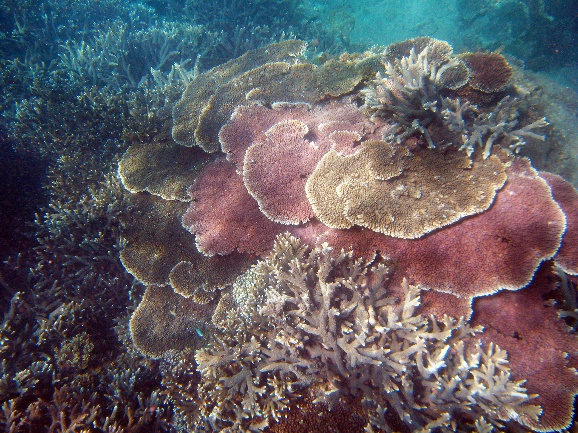 | 6.89 | .101 | 4.25 | 3.00 | 3.00 | 1.00 | 1.00 | 3.00 |
|  | 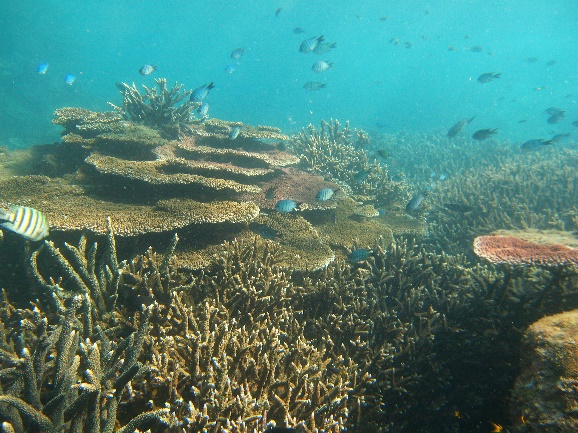 | 6.75 | .103 | 4.00 | 3.00 | 3.00 | 3.00 | 2.00 | 3.00 |
|  | 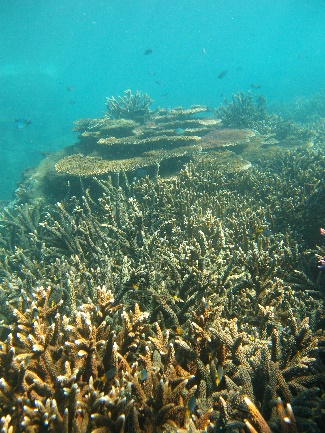 | 6.16 | .107 | 3.75 | 3.00 | 3.00 | 3.00 | 1.00 | 3.00 |
|  | 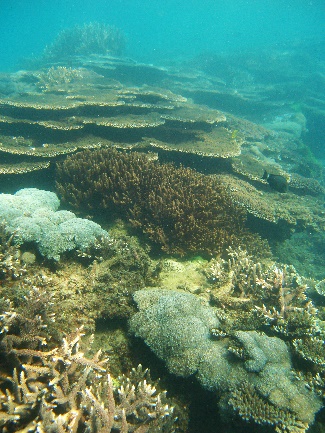 | 6.24 | .107 | 3.63 | 3.00 | 3.00 | 3.00 | 1.00 | 3.00 |
|  | 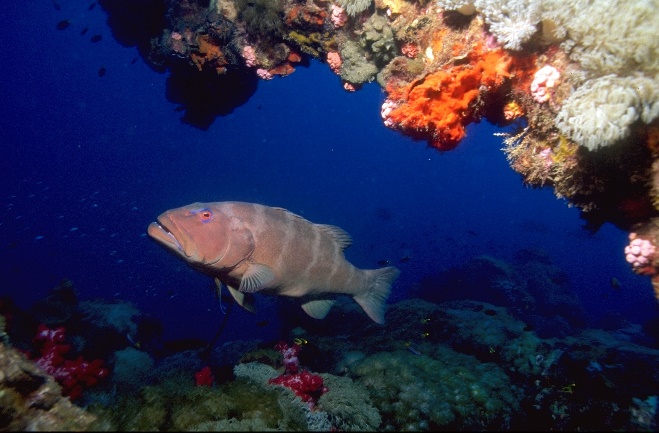 | 7.73 | .092 | 3.63 | 2.00 | 2.00 | 2.00 | 2.00 | 3.00 |
|  | 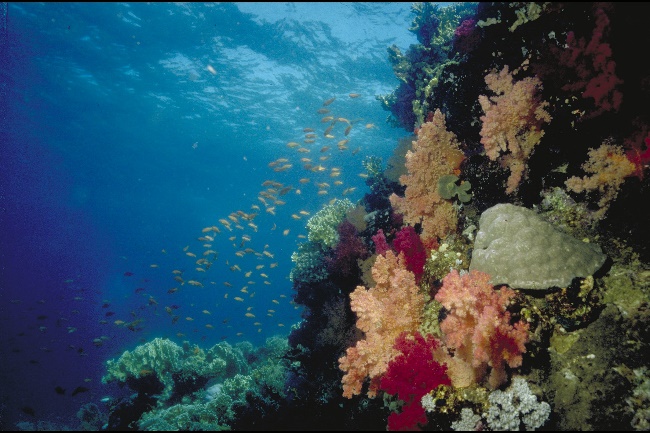 | 7.95 | .082 | 3.25 | 3.00 | 3.00 | 3.00 | 3.00 | 3.00 |
|  | 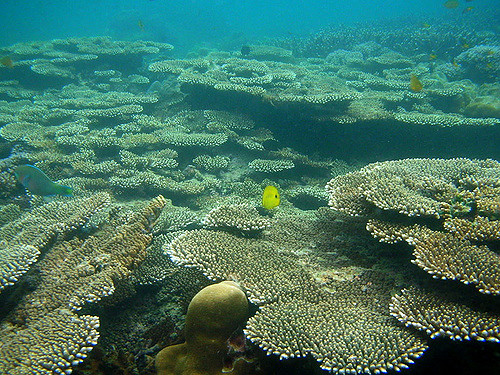 | 6.66 | .108 | 3.38 | 3.00 | 3.00 | 3.00 | 2.00 | 3.00 |
|  | 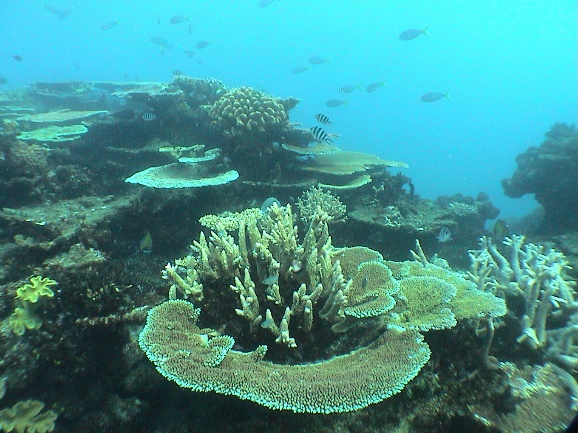 | 7.12 | .098 | 3.25 | 3.00 | 3.00 | 3.00 | 2.00 | 3.00 |
|  | 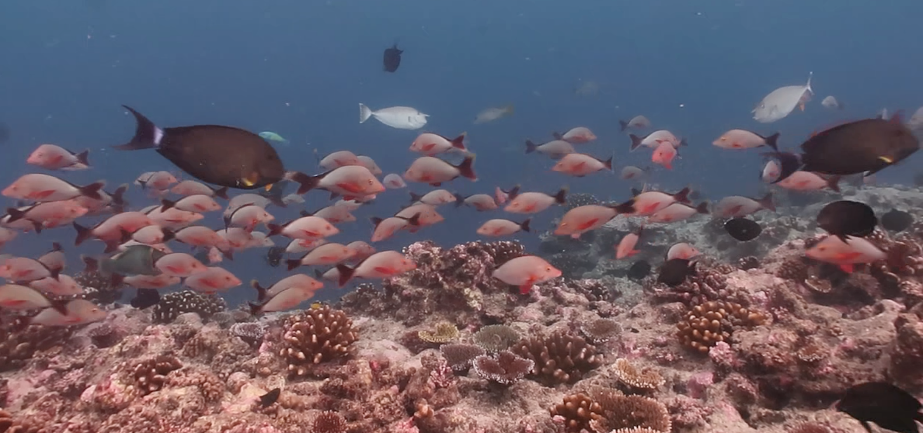 | 7.34 | .104 | 3.75 | 1.00 | 2.00 | 1.00 | 3.00 | 3.00 |
|  | 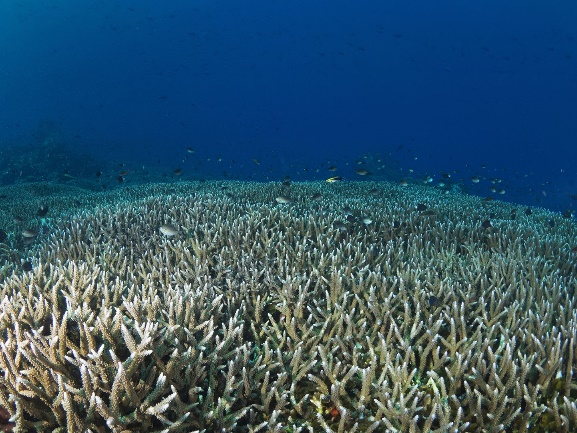 | 6.39 | .114 | 3.50 | 3.00 | 3.00 | 1.00 | 2.00 | 3.00 |
|  | 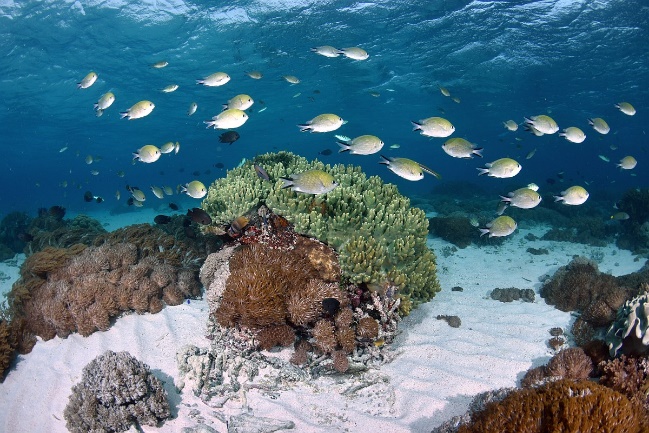 | 7.68 | .092 | 3.63 | 1.00 | 1.00 | 1.00 | 3.00 | 3.00 |
|  | 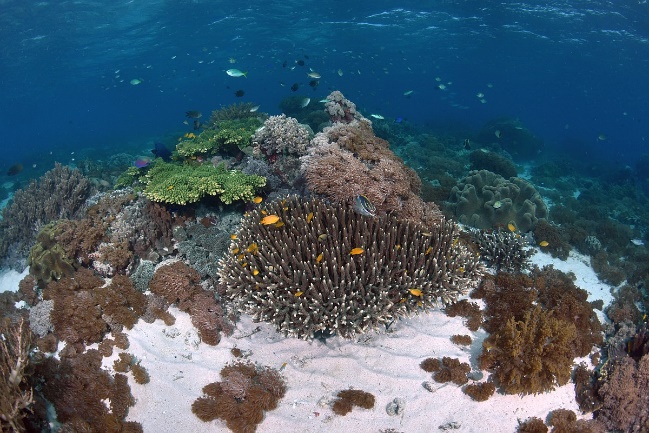 | 7.37 | .093 | 2.13 | 2.00 | 1.00 | 1.00 | 3.00 | 3.00 |
|  | 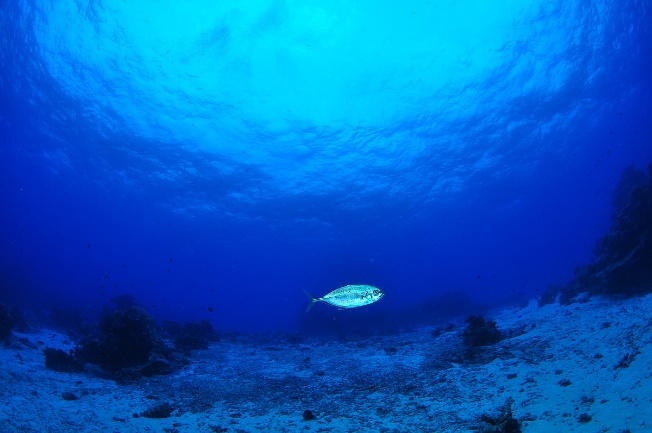 | 6.98 | .11 | 3.00 | 1.00 | 1.00 | 1.00 | 2.00 | 3.00 |
|  |  | 8.25 | .077 | 2.00 | 2.00 | 3.00 | 2.00 | 3.00 | 3.00 |
|  |  | 6.28 | .107 | 3.13 | 3.00 | 3.00 | 2.00 | 1.00 | 3.00 |
|  |  | 6.39 | .108 | 4.13 | 3.00 | 2.00 | 1.00 | 1.00 | 3.00 |
|  |  | 6.29 | .107 | 4.00 | 3.00 | 2.00 | 1.00 | 2.00 | 3.00 |
|  |  | 6.23 | .106 | 3.38 | 3.00 | 3.00 | 1.00 | 1.00 | 3.00 |
|  |  | 5.87 | .112 | 3.50 | 3.00 | 3.00 | 1.00 | 1.00 | 3.00 |
|  |  | 6.36 | .104 | 3.50 | 3.00 | 2.00 | 1.00 | 1.00 | 3.00 |
|  |  | 7.15 | .097 | 4.13 | 3.00 | 3.00 | 1.00 | 1.00 | 3.00 |
|  |  | 6.33 | .109 | 4.75 | 3.00 | 3.00 | 1.00 | 2.00 | 3.00 |
|  |  | 7.08 | .095 | 5.00 | 3.00 | 3.00 | 1.00 | 3.00 | 3.00 |
|  |  | 6.53 | .105 | 4.00 | 3.00 | 3.00 | 3.00 | 3.00 | 3.00 |
|  |  | 6.86 | .101 | 3.75 | 3.00 | 3.00 | 3.00 | 3.00 | 3.00 |
|  |  | 7.26 | .093 | 4.63 | 3.00 | 3.00 | 2.00 | 2.00 | 3.00 |
|  |  | 7.42 | .103 | 3.50 | 3.00 | 3.00 | 2.00 | 1.00 | 3.00 |
|  |  | 7.49 | .101 | 2.75 | 2.00 | 2.00 | 1.00 | 1.00 | 3.00 |
|  |  | 6.92 | .102 | 3.38 | 2.00 | 1.00 | 1.00 | 1.00 | 3.00 |
|  |  | 6.20 | .124 | 3.63 | 3.00 | 3.00 | 1.00 | 1.00 | 3.00 |
|  |  | 6.84 | .100 | 3.25 | 2.00 | 2.00 | 1.00 | 1.00 | 3.00 |
|  |  | 6.98 | .103 | 3.25 | 2.00 | 1.00 | 1.00 | 1.00 | 3.00 |
|  |  | 5.81 | .113 | 3.63 | 1.00 | 1.00 | 1.00 | 1.00 | 2.00 |
|  |  | 6.68 | .11 | 3.25 | 2.00 | 1.00 | 2.00 | 1.00 | 3.00 |
|  |  | 6.70 | .108 | 2.63 | 3.00 | 2.00 | 1.00 | 1.00 | 3.00 |
|  |  | 7.04 | .102 | 4.13 | 3.00 | 1.00 | 1.00 | 1.00 | 3.00 |
|  |  | 7.13 | .104 | 4.25 | 3.00 | 1.00 | 1.00 | 1.00 | 3.00 |
|  |  | 6.88 | .10 | 3.75 | 3.00 | 3.00 | 1.00 | 2.00 | 3.00 |
|  |  | 7.64 | .09 | 4.25 | 3.00 | 1.00 | 1.00 | 1.00 | 3.00 |
|  |  | 6.39 | .11 | 3.13 | 3.00 | 3.00 | 2.00 | 1.00 | 3.00 |
|  |  | 6.74 | .10 | 4.38 | 3.00 | 3.00 | 1.00 | 2.00 | 3.00 |
|  |  | 6.93 | .094 | 3.88 | 2.00 | 1.00 | 1.00 | 2.00 | 2.00 |
|  |  | 5.90 | .113 | 2.38 | 2.00 | 1.00 | 2.00 | 1.00 | 3.00 |
|  |  | 6.28 | .11 | 2.25 | 2.00 | 1.00 | 1.00 | 1.00 | 3.00 |
|  |  | 5.78 | .117 | 2.63 | 2.00 | 1.00 | 1.00 | 2.00 | 3.00 |
|  |  | 6.73 | .106 | 3.25 | 2.00 | 2.00 | 1.00 | 1.00 | 3.00 |
|  |  | 6.70 | .103 | 3.00 | 1.00 | 1.00 | 1.00 | 2.00 | 3.00 |
|  |  | 5.13 | .132 | 1.13 | 1.00 | 1.00 | 1.00 | 1.00 | 3.00 |
|  |  | 7.08 | .102 | 3.63 | 3.00 | 3.00 | 2.00 | 2.00 | 3.00 |
|  |  | 7.99 | .087 | 4.13 | 3.00 | 3.00 | 2.00 | 3.00 | 3.00 |
|  |  | 6.49 | .107 | 3.75 | 3.00 | 3.00 | 2.00 | 2.00 | 3.00 |
|  |  | 7.69 | .091 | 4.63 | 3.00 | 3.00 | 2.00 | 3.00 | 3.00 |
|  |  | 7.21 | .095 | 5.00 | 3.00 | 3.00 | 3.00 | 3.00 | 3.00 |
|  |  | 7.79 | .087 | 2.50 | 2.00 | 1.00 | 2.00 | 3.00 | 2.00 |
|  |  | 7.04 | .105 | 2.25 | 1.00 | 2.00 | 2.00 | 3.00 | 2.00 |
|  |  | 7.20 | .102 | 2.13 | 1.00 | 2.00 | 2.00 | 3.00 | 2.00 |
|  |  | 6.93 | .101 | 3.38 | 3.00 | 2.00 | 1.00 | 3.00 | 3.00 |
|  |  | 6.78 | .107 | 3.38 | 3.00 | 2.00 | 1.00 | 3.00 | 3.00 |
|  |  | 6.14 | .118 | 2.88 | 3.00 | 1.00 | 1.00 | 2.00 | 3.00 |
|  |  | 8.03 | .081 | 4.25 | 3.00 | 3.00 | 3.00 | 2.00 | 3.00 |
|  |  | 6.40 | .113 | 3.13 | 3.00 | 3.00 | 1.00 | 1.00 | 3.00 |
|  |  | 6.55 | .111 | 2.50 | 1.00 | 1.00 | 1.00 | 1.00 | 3.00 |
|  |  | 6.92 | .104 | 2.88 | 2.00 | 1.00 | 1.00 | 1.00 | 3.00 |
|  |  | 6.62 | .111 | 2.63 | 2.00 | 1.00 | 1.00 | 1.00 | 3.00 |
|  |  | 7.05 | .095 | 2.63 | 1.00 | 1.00 | 2.00 | 3.00 | 2.00 |
|  |  | 6.23 | .11 | 3.50 | 2.00 | 1.00 | 1.00 | 1.00 | 2.00 |
|  |  | 6.84 | .102 | 2.88 | 2.00 | 1.00 | 2.00 | 2.00 | 3.00 |
|  |  | 6.81 | .106 | 3.25 | 1.00 | 2.00 | 1.00 | 3.00 | 2.00 |
|  |  | 7.41 | .101 | 3.25 | 1.00 | 2.00 | 1.00 | 3.00 | 2.00 |
|  |  | 7.12 | .105 | 3.13 | 1.00 | 1.00 | 1.00 | 3.00 | 2.00 |
|  |  | 7.55 | .095 | 3.25 | 1.00 | 2.00 | 1.00 | 3.00 | 2.00 |
|  |  | 7.67 | .09 | 3.50 | 2.00 | 1.00 | 3.00 | 3.00 | 2.00 |
|  |  | 7.85 | .089 | 3.88 | 1.00 | 1.00 | 3.00 | 3.00 | 2.00 |
|  |  | 7.53 | .09 | 3.38 | 1.00 | 2.00 | 1.00 | 3.00 | 3.00 |
|  |  | 7.40 | .093 | 2.50 | 1.00 | 2.00 | 2.00 | 3.00 | 2.00 |
|  |  | 7.31 | .095 | 2.38 | 1.00 | 2.00 | 2.00 | 3.00 | 2.00 |
|  |  | 7.25 | .094 | 2.63 | 1.00 | 2.00 | 1.00 | 3.00 | 2.00 |
|  |  | 7.65 | .1 | 3.13 | 1.00 | 1.00 | 2.00 | 3.00 | 3.00 |
|  |  | 6.59 | .098 | 2.50 | 1.00 | 1.00 | 1.00 | 3.00 | 2.00 |
|  |  | 7.48 | .096 | 4.25 | 1.00 | 1.00 | 2.00 | 3.00 | 3.00 |
|  |  | 7.44 | .092 | 2.88 | 1.00 | 2.00 | 2.00 | 3.00 | 3.00 |
|  |  | 7.40 | .092 | 2.25 | 1.00 | 1.00 | 2.00 | 3.00 | 3.00 |
| 1. 1 |  | 7.43 | .102 | 3.75 | 3.00 | 1.00 | 2.00 | 3.00 | 3.00 |
|  |  | 5.92 | .111 | 3.38 | 3.00 | 1.00 | 2.00 | 1.00 | 2.00 |
|  |  | 5.79 | .11 | 3.25 | 2.00 | 1.00 | 1.00 | 3.00 | 3.00 |
|  |  | 7.96 | .088 | 3.38 | 1.00 | 2.00 | 2.00 | 3.00 | 3.00 |
|  |  | 7.22 | .091 | 3.00 | 1.00 | 1.00 | 2.00 | 3.00 | 3.00 |
|  |  | 7.35 | .093 | 3.88 | 2.00 | 1.00 | 1.00 | 3.00 | 3.00 |
|  |  | 6.60 | .112 | 2.75 | 3.00 | 1.00 | 1.00 | 1.00 | 3.00 |
|  |  | 7.28 | .095 | 3.25 | 1.00 | 1.00 | 2.00 | 3.00 | 2.00 |
|  |  | 7.89 | .079 | 3.88 | 2.00 | 2.00 | 2.00 | 3.00 | 3.00 |
|  |  | 6.79 | .107 | 4.00 | 3.00 | 2.00 | 3.00 | 2.00 | 3.00 |
|  |  | 7.40 | .094 | 2.38 | 3.00 | 3.00 | 3.00 | 3.00 | 3.00 |
| 1. 1 |  | 7.43 | .102 | 3.75 | 3.00 | 1.00 | 2.00 | 3.00 | 3.00 |
