# Supplementary material for: Identifying indicators of aesthetics in the Great Barrier Reef for the purposes of management
Source: PLoS One. 2019 Feb 20;14(2):e0210196. doi: 10.1371/journal.pone.0210196 (PMC6382102; doi:10.1371/journal.pone.0210196)
Supplement: S2 File — (DOCX) [file pone.0210196.s002.docx]

## Supporting Information 2.

## The verbatim list of indicators of aesthetic values, how they have been thematically coded, and categorised into the final list of 12 indicators.

| **verbatim indicators of aesthetic values** | **thematic indicators of aesthetic values** | **category** |
| --- | --- | --- |
| clear blue water | clear-water | clear-water |
| colour | colour | colour |
| movement | movement | experience |
| depth of scale | depth | composition |
| complexity | complexity | composition |
| structure | structure | composition |
| symmetry | symmetry | composition |
| composition of subject against background: Fibonacci | composition | composition |
| An absence of people | no-people | naturalness |
| green | green | colour |
| white sand | white sand | colour |
| inspired by the opportunity to explore | opportunity | experience |
| Feng Shui | Feng-Shui | composition |
| Sense of space | space | composition |
| clear water | clear-water | clear-water |
| colourful coral | coral-colour | coral |
| lots of fish | fish-abundance | fish |
| different sized fish | fish-diversity | fish |
| coral cover | coral-cover | coral |
| coral diversity | coral-diversity | coral |
| clear water | clear-water | clear-water |
| habitat complexity | habitat-complexity | composition |
| blue | blue | colour |
| green | green | colour |
| healthy coral | healthy | healthy |
| healthy fish | healthy | healthy |
| no rubbish | clean | clear-water |
| clean water | clear-water | clear-water |
| no toxins in the water | safe | clear-water |
| alive | healthy | healthy |
| natural function | naturalness | naturalness |
| natural diversity | naturalness | naturalness |
| Biodiversity | biodiversity | composition |
| flourishing | flourishing | healthy |
| visual heterogeneity | diversity | composition |
| structural heterogeneity | structural heterogeneity | comlexity |
| big fish | big-fish | fish |
| integrity | integrity | healthy |
| variety of fish | fish-diversity | fish |
| massive coral | massive--coral | coral |
| naturalness | naturalness | naturalness |
| vibe | vibe | experience |
| sensation | sensation | experience |
| diversity | diversity | composition |
| different sizes | size-diversity | composition |
| different shapes | shape-diversity | composition |
| colour | colour | colour |
| movement | movement | experience |
| tiny animals | tiny -things | tiny-things |
| feeling of where I want to be | destination | experience |
| clear water | clear-water | clear-water |
| coral structure | coral-structure | coral |
| physical diversity | physical-diversity | composition |
| white sand | white-sand | colour |
| turquoise water | turquoise-water | colour |
| beaches | beach | naturalness |
| pattern | pattern | composition |
| forested island | forested-island | naturalness |
| biophyscial diversity | structural heterogeneity | composition |
| colour of the water | colour | colour |
| colour of the fish | fish-colour | fish |
| iconic fish | iconic | charismatic |
| the sea and the sky together | sky-and-sea | colour |
| interactions with nature | connection | experience |
| feeling of destination reached | destination | experience |
| the experience of the wind and the waves | the experience of the wind and the waves | experience |
| pristine places | pristine | naturalness |
| water quality | water-quality | clear-water |
| naturalness | naturalness | naturalness |
| healthy system | healthy | healthy |
| diversity | diversity | composition |
| Biodiversity | biodiversity | composition |
| Outstanding Universal Value criteria | OUV | charismatic |
| coral cover | coral-cover | coral |
| clear water | clear-water | clear-water |
| huge expanse | expanse | composition |
| coral diversity | coral-diversity | coral |
| diversity of form | structural-diversity | composition |
| coral colour | coral-colour | coral |
| fish | fish | fish |
| meagfauana | megafauna | charismatic |
| aerial view | aerial-view | aerial |
| naturalness | naturalness | naturalness |
| clear water | clear-water | clear-water |
| movement of fish | movement | experience |
| colour of fish | fish-colour | fish |
| reef drop offs | drop-offs | experience |
| sound of fish munching coral | fish-munching-sound | experience |
| massive coral | massive-coral | coral |
| tiny things | tiny-things | tiny-things |
| coral design | coral-design | coral |
| diversity of structure | structural-diversity | composition |
| nature in action | naturalness | naturalness |
| lovely weather | sunshine | experience |
| blue | blue | colour |
| colour | colour | colour |
| channels to swim in | drop-offs | experience |
| aerial view | aerial-view | aerial |
| lack of disease | healthy | healthy |
| emotional connections | connections | experience |
| clean water | clean water | clear-water |
| Biodiversity | biodiversity | composition |
| coral | coral | coral |
| birds | birds | charismatic |
| fish | fish | fish |
| mammals | mammals | charismatic |
| naturalness | naturalness | naturalness |
| blue | blue | colour |
| green | green | colour |
| white sand | white sand | colour |
| people enjoying themselves | recreation | recreation |
| no rubbish | clean | clear-water |
| biodiversity of coral | coral-diversity | coral |
| topography | topography | composition |
| coral health | healthy | healthy |
| diverse ecosytem | diverse ecosytem | composition |
| healthy ecosystem | healthy | healthy |
| no disease | healthy | healthy |
| diversity of fish | fish-diversity | fish |
| diversity of invertebrates | invertebrates | tiny-things |
| coral cover | coral-cover | coral |
| white sand | white-sand | colour |
| blue | blue | colour |
| green | green | colour |
| aerial view | aerial-view | aerial |
| colour | colour | colour |
| expansive view | space | experience |
| rainforest and reef together | rainforest-reef | naturalness |
| clear water | clear-water | clear-water |
| diversity of coral | coral-diversity | coral |
| massive coral | massive-coral | coral |
| different forms | structural-diversity | composition |
| movement | movement | experience |
| schools of fish | fish-schools | fish |
| people enjoying themselves | recreation | recreation |
| tiny things | tiny-things | tiny-things |
| alive | life | healthy |
| fringing reefs | fringing-reefs | coral |
| form | form | composition |
| structure | structure | composition |
| pattern | pattern | composition |
| camouflague | camouflague | tiny-things |
| complexity | complexity | composition |
| sounds | sound | experience |
| tiny things | tiny-things | tiny-things |
| school of fish | fish-schools | fish |
| healthy | healthy | healthy |
| aerial view | aerial-view | aerial |
| blue | blue | colour |
| turquoise water | turquoise-water | colour |
| white sand | white-sand | colour |
| coral cover | coral-cover | coral |
| invertebrates | invertebrates | tiny-things |
| small things | tiny-things | tiny-things |
| sessile organisms | sessile | tiny-things |
| ascidians | ascidians | tiny-things |
| reef wall | drop-offs | experience |
| naturalness | naturalness | naturalness |
| beaches | beach | naturalness |
| sound | sound | experience |
| smell | smell | experience |
| feel | feel | experience |
| taste | taste | experience |
| natural beauty | naturalness | naturalness |
| aerial views | aerial-view | aerial |
| rainforest on the beach | rainforest-reef | naturalness |
| charismatic fauna | charismatic-fauna | charismatic |
| whales | whales | charismatic |
| turtles | turtles | charismatic |
| seabrids | seabirds | charismatic |
| massive schools of fish | fish-schools | fish |
| recreational use | recreation | recreation |
| small boats | boats | recreation |
| divers and snorkellors | recreation | recreation |
| clear water | clear-water | clear-water |
| big fish | big-fish | fish |
